# Enhanced Binding of (3-Aminopropyl)triethoxysilane to Polymer Brush-Coated Surfaces by Controlled Activation: Degradation, Activation, and Functionalization

**DOI:** 10.1021/acsomega.5c06525

**Published:** 2025-08-29

**Authors:** Nadezda Prochukhan, Arantxa Davó-Quiñonero, Alberto Alvarez-Fernandez, Pravind Yadav, Sergey Beloshapkin, Abbie Jane Keegan, Ross Lundy, Michael A. Morris

**Affiliations:** † School of Chemistry, CRANN and AMBER Research Centres, 8809 Trinity College Dublin, College Green, Dublin 2, Ireland; ‡ BiOrbic — Bioeconomy SFI Research Centre, University College Dublin, Belfield, Dublin 4, Ireland; § University of Alicante, Carretera San Vicente del Raspeig s/n, Alicante 03690, Spain; ∥ Department of Chemical Sciences, Bernal Institute, 607418 University of Limerick, Limerick V94 T9PX, Ireland

## Abstract

Polymer brushes are widely used to control the surface chemistry of substrates and selectively block or allow metal deposition on samples. Polymer brushes have advantages such as the possibility of conformal coatings and chemical binding to surfaces, thus improving stability. Despite progress in the application of polymer brushes such as poly­(methyl methacrylate) (PMMA) and polystyrene (PS) for area-selective deposition (ASD), brush removal and activation have not been widely investigated. Herein, we present a method to fabricate a stacked brush APTS-PS or APTS-PMMA composite and investigate brush removal with UV ozone (UVO) treatment and oxygen plasma ashing. We believe this work will lead to improved processing of brushes and allow for the advancement of the ASD field.

## Graphical abstract

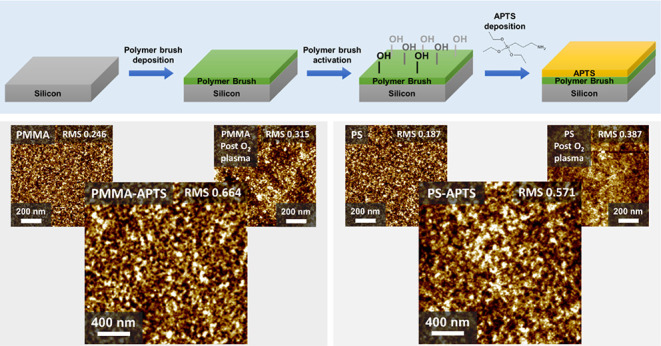

## Introduction

The drive for miniaturization in the microelectronics industry over the past 30 years, combined with the growing interest in flexible and stretchable electronics, has led to a constant need for more precise and simpler fabrication methodologies for the production of thin films with controllable dimensions, shapes, and densities. Thus, achieving nanometric precision in shaping metallic thin films is crucial for the fabrication of various conductive components, such as interconnects, circuits, and contact pads, playing a vital role in numerous applications, including wearable displays, medical implants, high-power electronics, and microelectromechanical systems.

Chemical vapor deposition (CVD) and physical vapor deposition (PVD) are among the most common and well-established methods for thin-film deposition. Thus, while CVD involves a chemical reaction of reactive gases that results in the growth of a thin film on the substrate, PVD relies on the deposition of a vaporized material, through methods such as sputtering and evaporation, which results in the condensation of a thin film on the substrate. Both CVD and PVD are known for their ability to provide precise control over the thickness and purity of the thin films produced. However, challenges such as the formation of byproducts during the growth process (in the case of CVD) or having a limited coating area (for PVD) have contributed to the search for alternative methods for thin-film production.

As an alternative to these two well-established techniques (and their derivatives), one of the most important alternatives in the fabrication of highly controllable thin-film materials is polymer-assisted deposition. It involves the incorporation of metallic precursors into a polymer film, which acts as a sacrificial template for the deposition of the thin film material. In this sense, polymer brush chemistry has gained visibility as a versatile technique to modify surfaces for area-selective deposition (ASD).^1–3^ Polymer brushes offer the possibility to adjust the surface energy of flat and complex surfaces^1^ as well as to pattern specific areas by providing areas of the substrate that block or activate the substrate to specific chemistries.^4,5^ The term polymer brush is rather loosely used; we define a polymer brush as a low molecular weight polymer that has termination chemistries that allow chemical bonding to the substrate, thus offering long-term stability. Furthermore, the brushes can adapt to and adopt the surface roughness of the substrate.^2^ Thus, the brushes can act as substrates themselves. Multiple studies report polymer brush coatings and subsequent infiltration with metal ions during ASD. Potential treatments, such as UV/ozone (UVO), have been used to remove the polymer, leaving behind a pristine metal oxide.^2,3,5–7^ Therefore, it is important to understand how polymer brushes are removed under harsh conditions, such as UVO or oxygen plasma treatment.

The UVO and oxygen plasma treatments are both known to produce hydrophilic surfaces by removing the adsorbed hydrocarbons and cleaving bonds such as C–C linkages, enhancing oxidation and hydroxylation.^8–10^ Here, polymer surfaces oxidize, and gases such as CO, CO_2_ and H_2_O are released, which result in polymer degradation at the surface or bulk depending on factors such as time, temperature, and so on. For polymer brush films (typically a few nm in thickness), full polymer removal can be achieved with those techniques within minutes. Previous studies report time scales of hours for polymer removal;^1–3,11,12^ however, we demonstrate that only minute scales are required for the complete removal of widely used ultrathin polymer brushes, namely polystyrene (PS) and poly­(methyl methacrylate) (PMMA). It is important to note that the metal-infiltrated polymer brushes can exhibit different degradation time scales (typically longer times) if strong intermolecular forces exist between the brushes and metal moieties, for instance. The metal can also protect the brush from oxidation.^13^ Thus, we expect that longer UVO or plasma treatment times may be beneficial as a precaution in cases of metal infiltration into polymer brushes.

There is significant research toward surface functionalization, whereby surface chemistries allow selective binding of molecules. Polymer brushes may be highly effective through design or by modification after surface binding. We focus on polymer brushes where the brush exposure time to UVO or oxygen plasma can be used to modify the brush surfaces via UVO or plasma treatment. Here, we demonstrate that brush-coated surfaces can be adapted to functionally bind chemicals, for example, OH-terminated ligands in vapor or liquid form. Herein, a common ligand, (3-aminopropyl)­triethoxysilane (APTS), is employed to demonstrate the possibility of building a stacked functional layer atop the brushes. APTS contains hydrolyzable alkoxy groups, which make it an excellent molecule for chemisorption to hydroxyl-terminated surfaces.^14^ APTS is commonly used because it can be used to bind various biomolecules, e.g., bioassay and chromatographic separation.^14^ The APTS brush layer was altered to produce either a blocking layer or an active layer, as desired. In this work, we show brush degradation as well as surface activation for APTS binding by the combination of atomic force microscopy (AFM), scanning electron microscopy (SEM), contact angle (CA), and time-of-flight secondary ion mass spectrometry (ToF-SIMS) analyses. We expect that our findings showcase the versatility of polymer brushes and provide insight into the degradation time scales under widely used UVO and oxygen plasma methods. Overall, the study showcases different methodologies for functionalization and characterization of polymeric surfaces, which can be applicable to irregular or rough surfaces whereby conventional characterization methods may be difficult to apply. Specifically, the advantage of this study lies in the facile characterization process as well as the fast time scales for polymer functionalization.

## Experimental Section

### Materials

The chemicals (analytical grade) used in this report were purchased from Sigma-Aldrich. Hydroxy-terminated polystyrene (PS–OH) (number-average molecular weight, *M*_n_ = 10 kg mol^–1^, *M*_w_/*M*_n_ = 1.09, Mw: weight-average molecular weight), hydroxy-terminated poly­(methyl methacrylate) (PMMA–OH) (*M*_n_ = 6.3 kg mol^–1^, *M*_w_/*M*_n_ = 1.06), and polystyrene (PS) (*M*_n_ = 17.0 kg mol^–1^, *M*_w_/*M*_n_ = 1.03) were purchased from Polymer Source Inc. and were used without further purification. Silicon <100> p-type wafers with a native oxide layer were used as substrates. (3-Aminopropyl)­triethoxysilane (APTS), acetone (ACS reagent, ≥ 99.5%), toluene (CHOMASOLV, for HPLC, 99.9%), and ethanol (dehydrated, 200 proof) were purchased from Sigma-Aldrich and used without further purification unless otherwise stated.

PMMA pellets were purchased from Altuglas (BS572), with a number-average molecular weight, *M*_n_ = 440.5 kg mol^–1^, *M*_w_/*M*_n_ = 1.04, Mw: weight-average molecular weight (Mw) was measured using gel permeation chromatography (GPC).

### Polymer Brush Preparation

Brush optimization was conducted according to the method reported by Lundy et al.^2,3^ Silicon substrates were cleaned in toluene by sonication for 20 min and dried with N_2_ gas. Substrate surfaces were further cleaned and hydroxyl functionalized via oxygen plasma for 3 min (40 kHz, 120 W, Diener PICO Barrel Asher). The substrates were spin coated (3000 rpm, 25 s, 5 s ramp) with 0.2 wt % PS–OH brush solution in toluene (stirred for 1 h). The polymer brush was annealed on a hot plate at 200 °C for 5 min. Subsequently, substrates were sonicated in toluene for 20 min twice to remove any physically adsorbed layers and dried in dry N_2_ gas afterward. The same process was repeated using 0.6 wt % PMMA–OH brush solution in toluene.

### Brush Activation Optimization

PMMA and PS brushes were subjected to UVO treatment (PSD Pro Series, Digital UV Ozone System, Novascan) from 10 s to 3 h to study contact angle (CA) changes and the onset of degradation. The samples were positioned 4 cm from the UV lamp (mercury lamp, output current 0.8 – 0.95 A, power 65 – 100 W, emission at 184.9 and 253.7 nm).^15^ Separate PS and PMMA brush samples were also exposed to different RF powers of oxygen plasma ashing for 1 min (10% RF power or 20 W, 20% RF power or 40 W, and 60% RF power or 120 W), and the water CAs were studied.

### Brush Functionalization with APTS

The optimization of the vapor deposition conditions was conducted on plasma-activated PMMA powder from Altuglas. The pellets were dissolved in acetone, and subsequently, ethanol (nonsolvent for PPMA) was added to produce an amorphous porous PMMA mass (membrane-like). The solvents were evaporated in a fume hood at room temperature (20 ± 1 °C).

This allowed the PMMA to be ground into finer powder using a coffee grinder equipped with a ceramic burr to prevent sample degradation (HK2901, KitchenPRO, dimensions: 6.35 × 6.1 × 22.61 cm^3^; 204.12 g). The ground powder was exposed to UVO treatment and oxygen plasma (ashing, 40W, 1 min). The activated powder was placed in an open vial inside a jar with another open vial containing 50 μL of APTS and sealed before being placed inside a vacuum oven at 110 °C at 200 mbar over a period of 4 to 10 h. Quantification of APTS was conducted via the Ninhydrin test.^16^ The APTS-PMMA sample of interest (0.5 g) was immersed in a sealed vial containing the Ninhydrin reagent (1 mL) and water (2 mL). The solutions with the samples were heated to 70 °C for 15 min. A similar procedure was performed for pure APTS to construct a calibration curve for known APTS concentrations up to 5 μM. The solid samples were extracted by filtration, and the solutions were diluted to a volume of 100 mL using volumetric flasks. Absorbance at 570 nm was measured, and the APTS functionalization degree was quantified from the standard calibration plot. APTS functionalization was also characterized via XPS and FTIR.

Brush samples (PS and PMMA) and blank silicon wafers were subjected to an O_2_ plasma ashing treatment for 1 min at 40 W. Subsequently, the plasma-activated brushes were used in APTS vapor deposition experiments in a vacuum oven for 7 h as described above. The samples were further rinsed in toluene to remove physiosorbed material and yield only chemically bonded structures. Where applicable, values are reported as the sample mean value ± 1s, where s stands for sample standard deviation.

### Characterization

**UV–vis spectroscopy** was performed using a PerkinElmer/Lambda 35 spectrophotometer in the 300 to 700 nm range, with a step of 1 nm at a scan speed of 480 nm min^–1^.

**Grazing-angle attenuated total reflection infrared (GAATR IR)** spectra were performed using a Harrick VariGATR accessory coupled with a Ge hemispherical ATR crystal. The spectra were recorded with an unpolarized Nicolet iS50 spectrometer (ThermoFisher Scientific) of an incidence angle of 65°, 128 scans per experiment, and a resolution of 8 cm^–1^.

**Spectroscopy ellipsometry (SE)** measurements were carried out on a Semilab SE-2000 ellipsometer (within the photon energy range of 1.3–4.5 eV). All ellipsometric data analysis was performed with SEA software using the Tauc–Lorentz dispersion model fitting.

**Dynamic contact angle (CA)** measurements (custom-built system) were recorded on five different regions of each sample using a high-speed camera (60 Hz sampling rate) to capture the water advancing CAs. The procedure is outlined by Lundy et al.^3^ CAs were quantified on ImageJ using “drop snake” plugin.^17,18^ PS homopolymer and PMMA homopolymer were used to fabricate pure PS and PMMA substrates. The powders were dry-pressed into disc-shaped pellets ≈2 mm thick at a pressure of 350 MPa in a 13 mm diameter steel pellet die (Specac, 13 mm evacuable pellet die). The as-pressed pellets were exposed to toluene solvent vapor until a smooth, mirror-like surface was produced. APTS melt was deposited onto Si substrates to produce flat substrates for water CA measurements.

**Scanning electron microscopy** (SEM, Zeiss Ultra Plus) was performed at an accelerating voltage of 2 kV and a working distance of 4–5 mm using a 30 μm aperture.

**Atomic force microscopy** (AFM, Park Systems, XE7) was operated in NCM (noncontact mode) under ambient conditions using a silicon microcantilever probe tip (force constant of 42 N m^1–^).

**Time-of-flight secondary ion mass spectrometry** (ToF-SIMS, ION-TOF GmbH). For high lateral resolution and imaging as well as high mass resolution surface spectroscopy. This instrument is equipped with a 30 keV, three-lens BiMN cluster liquid metal ion gun. All analyses were performed with Bi^+^ primary ions in the positive ion mode and with a cycle time of 95 μs. The field of view for analysis was 300 × 300 μm^2^ with each image containing 128 × 128 pixels; each of these pixels contains the entire mass spectrum. The analysis time was approximately 150 to 180 s, set to 5 × 10^12^ primary ions cm^–2^. This specific setup allows acquiring images with an expected lateral resolution of 3–5 μm. The peaks in each spectrum were normalized by total ion intensity and mean-centered with respect to small hydrocarbon ions.^19,20^ The spectra were analyzed with SurfaceLab 5, and the peak areas were normalized by the total ion intensity. The extracted matrix of ions and corresponding areas was mean-centered for principal component analysis (PCA), which was conducted in PAST.^21^ PCA and spectrum extraction were also conducted on a per sample basis in Python using the pySPM package.^22^ The pySPM package was also used to construct 2D maps and overlay images of selected ions on a per-sample basis.

## Results and Discussion

### Activation of Polymer Brushes

The brushes used in this work were prepared according to Lundy et al.^3^ The chemical structures of the PS and PMMA brushes, as well as APTS, are shown in Figure 1. The OH- functional groups graft to the surface of the silicon substrate. In the case of APTS, the alkoxy groups hydrolyze to yield hydroxyls. These hydroxyl groups can undergo condensation reactions as shown in Figure 1.^14^

**Figure 1:**
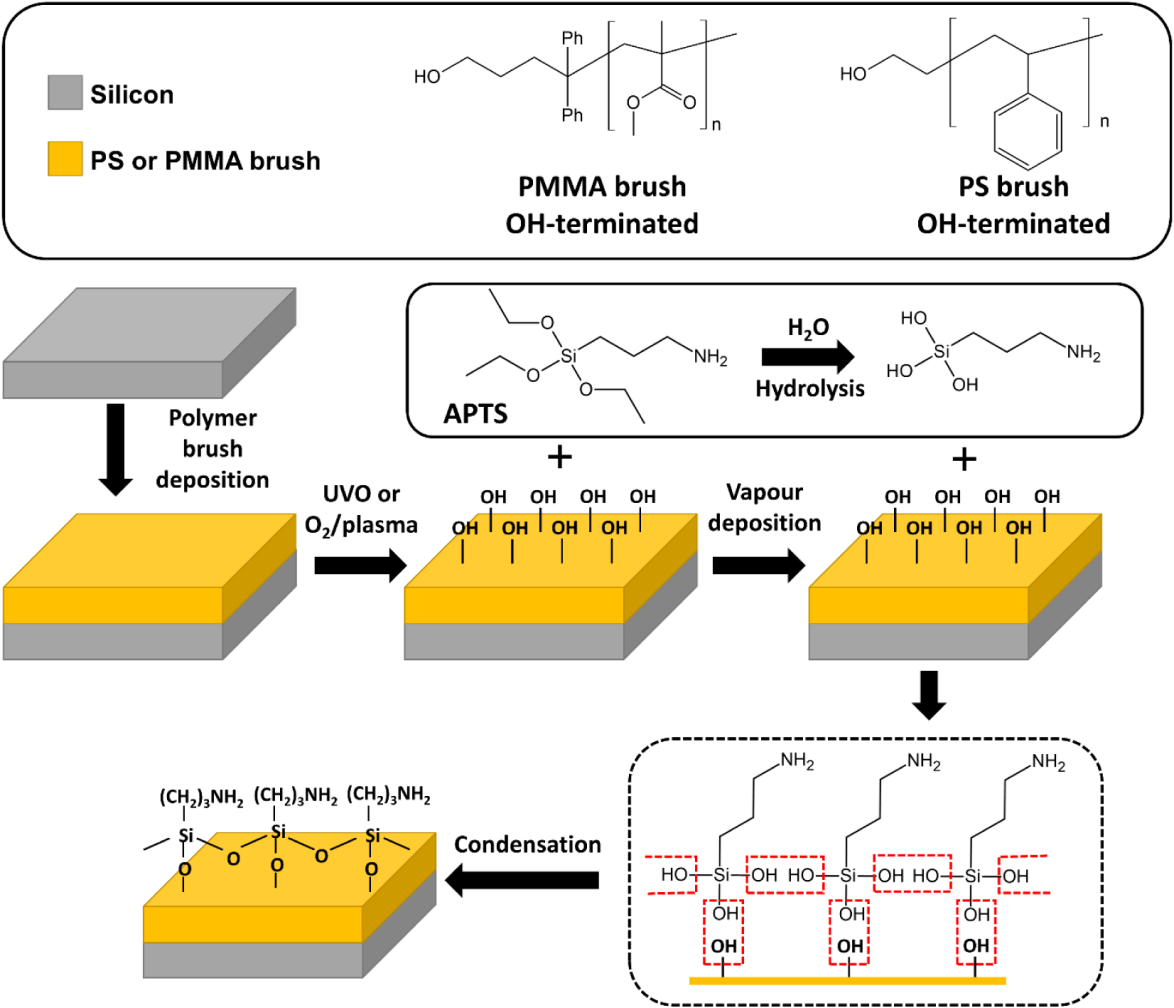
General scheme of the deposition process of the PS and PMMA brushes with subsequent APTS deposition.

The initial UVO and plasma treatment optimizations were conducted on PS and PMMA brushes and analyzed by CA and AFM. In the case of PMMA brushes, the UVO treatment leads to rapid brush degradation, as can be seen from Figure 2A. Thus, the CA measurements after 45 s UVO show a value close to that of a fully hydroxylated surface (63.79 ± 0.96°). However, after 60 s of treatment, the angle increases again, which is a sign of surface degradation as the surface roughness increases. On the other hand, plasma treatment at 20% RF power (or 40 W RF power) also shows a considerable reduction in CA (43.29 ± 3.07°), which is associated with surface cleaning and hydroxylation (Figure 2B). It is important to note that the water CAs of the activated brush samples remained at the measured values only for up to 1–2 days before beginning to increase. Thus, the samples were used immediately postactivation.

**Figure 2:**
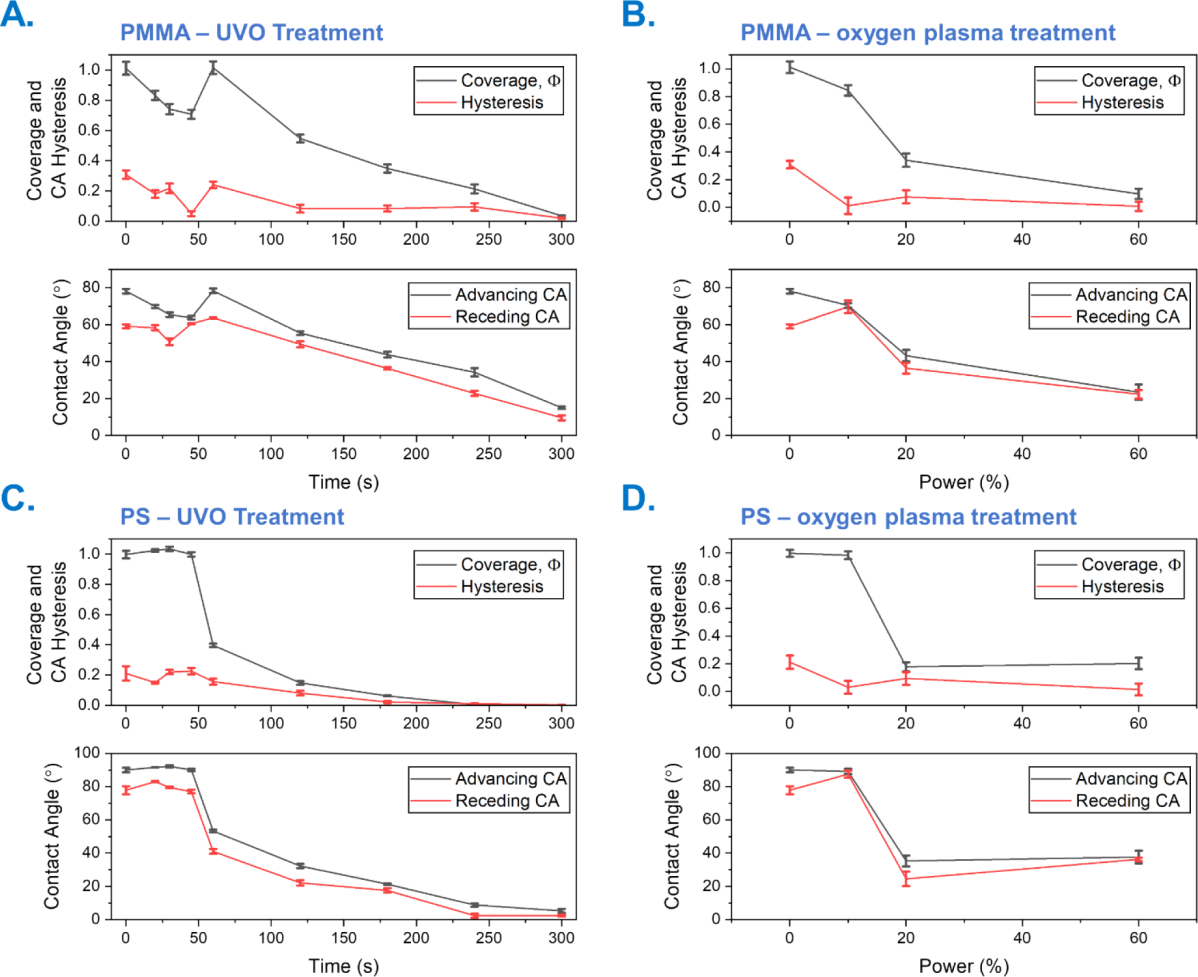
Polymer brush exposure to UVO treatment as a function of time for (**A**) PMMA and (**C**) PS brushes to 1 min oxygen plasma treatment as a function of plasma ashing power for (**B**) PMMA and (**D**) PS brushes. The advancing and receding water CAs on PMMA and PS brushes and associated coverage and CA hystereses are displayed.

In order to confirm this trend, CA hysteresis was calculated for all samples. The hysteresis between the advancing and receding CAs shows whether the CAs are different and the extent of said difference. A large value indicates an increase in surface roughness; i.e., droplet pinning occurs due to pinholes in the surface. Following this idea, CA hysteresis was calculated as shown in eq 1.^23^

1 $$\mathrm{Hysteresis}=\cos\left(\theta_{\mathrm{receding}}\right)-\cos\left(\theta_{\mathrm{advancing}}\right)$$

where θ_receding_ is the water receding CA in radians and θ_advancing_ is the advancing water CA in radians. The cosine formula is used to express the relationship between angles in radians on a comparable scale. In this case, the maximum expected advancing CA is approximately 90° for PS, and the minimum possible receding CA is close to 0°; thus, the hysteresis based on the cosine formula should range from 0 to 1. As shown in Figure 2, while for 45 s and over 60 s UVO treatments, PMMA surfaces show hysteresis values consistently below 0.2, which is typical of smooth surfaces, for the 60 s UVO treatment, the hysteresis value goes above 0.2, confirming the surface degradation and the increase in surface roughness. On the contrary, all samples treated with plasma show a low hysteresis, indicating a smooth surface.

For the PS brush, similar results are observed, as seen in Figure 2. The PS brushes show a slower degradation than PMMA under UVO, consistent with the differences in the chemical structures of the brushes.^9,24,25^ In any case, after 60 s of UVO treatment, a considerable reduction in CA is observed (Figure 2C). Similarly, plasma at 20% power causes the water CA to drop to around 40° (Figure 2D), indicating the degradation of the polymeric brush. It is important to note here that even if after 10% power plasma treatment the hysteresis drops considerably (indicating smoothening of the surface), only after 20% plasma power a reduction in the CA is observed, which is related to a more hydrophilic film corresponding to an activated polymer surface.

Another important factor to consider when evaluating the degradation of polymeric brushes is the surface coverage. This can be calculated using the Cassie–Baxter model,^2^ shown in eq 2.

2 $$\Phi =\left(\frac{\cos\theta_{\mathrm{Brush}}}{\cos\theta_{SiO_{2}}}-1\right)/\left(\frac{\cos\theta_{\max}}{\cos\theta_{SiO_{2}}}-1\right)$$

where Φ indicates the surface coverage, with values ranging from 0 (no coverage) to 1 (full coverage), θ_Brush_ is the advancing water CA at a given PS or PMMA concentration, θ_SiO2_ is Si substrate CA after the same pretreatment as the brush samples, and θ_max_ is the CA of the PS and PMMA pellet, respectively. Following this, θ_SiO2_ was established at 6.63 ± 0.39°, while obtained values of θ_max_ evolved from 90.18 ± 0.48° (PS), to 77.64 ± 1.46° (PMMA). In the case of the PMMA and following eq 2 and the values previously provided, a clear drop in the surface coverage values obtained is detected after 45 s of UVO treatment and 20% plasma, consistent with surface activation and/or degradation for PMMA. The jump in the surface coverage at 60 s could indicate the full removal of the upper layers of the PMMA brush. Regarding the PS, a similar trend is observed, with a clear reduction in the surface coverage values after 60 s of UVO treatment and 20% plasma. Both the CA and the surface coverage analyses show the full degradation of the PS and PMMA polymeric brushes after 300 s of UVO treatment and 1 min of 60% power plasma.

The AFM data in Figure 3 explains the increase in CA for PMMA at 60 s UVO, i.e., it is consistent with the Wenzel equation:^26^

3 $$\theta_{\mathrm{apparent}}=r\cos\theta$$

where the apparent or measured CA (θ_apparent_) in radians is related to the actual CA (θ) in radians given a smooth surface, *r* is simply the roughness factor defined as the surface area divided by the geometric surface area.^26^ For 60 s UVO-treated PMMA, the *r* value is approximately 0.0265, whereas for 45 s sample, *r* is approximately 0.001, as measured from the AFM images. Therefore, the increased roughness upon degradation onset is explainable for PMMA. The 3 min UVO-treated PS brush has only a slightly larger *r* value of ∼0.0034 compared to the 1 min treated sample (r of ∼0.001). Thus, a closer look at the AFM micrographs is essential to establishing optimal conditions for both brushes.

**Figure 3:**
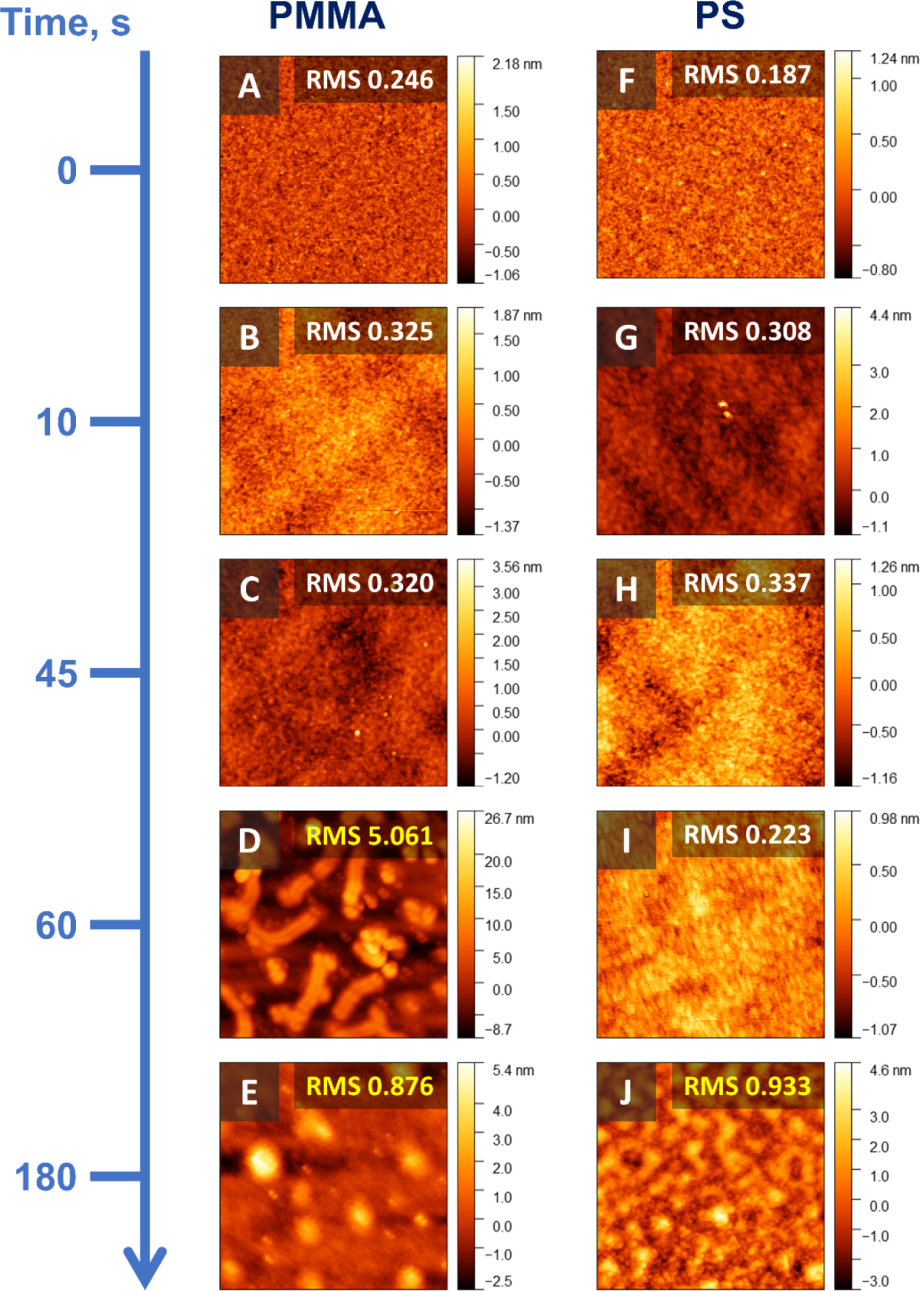
AFM micrographs (1 × 1 μm^2^) of UVO-treated PMMA (A–E) and PS (F–J) brushes with associated UVO times and RMS roughness values are indicated in the insets.

The AFM analysis of the local roughness (Figure 3) pinpoints the exact surface degradation onset. For PS brushes, the optimal time for UVO treatment is ∼1 min (i.e., the root-mean-square (RMS) roughness is low, < 1), while for PMMA brushes, at 1 min, the film noticeably degrades (RMS roughness >1). The optimal UVO activation time for PMMA is thus chosen to be 45 s. The AFM analysis is thus consistent with the CA data.

The AFM analysis after plasma treatment does not indicate whether the brush degraded or not (RMS roughness values <1); however, the surface remains smooth, as seen for both PMMA and PS brushes in Figure 4. In this case, whereby a smooth film is desirable, plasma treatment is a preferential method over UVO treatment, as the degree of surface roughness is preserved or even improved.

**Figure 4:**
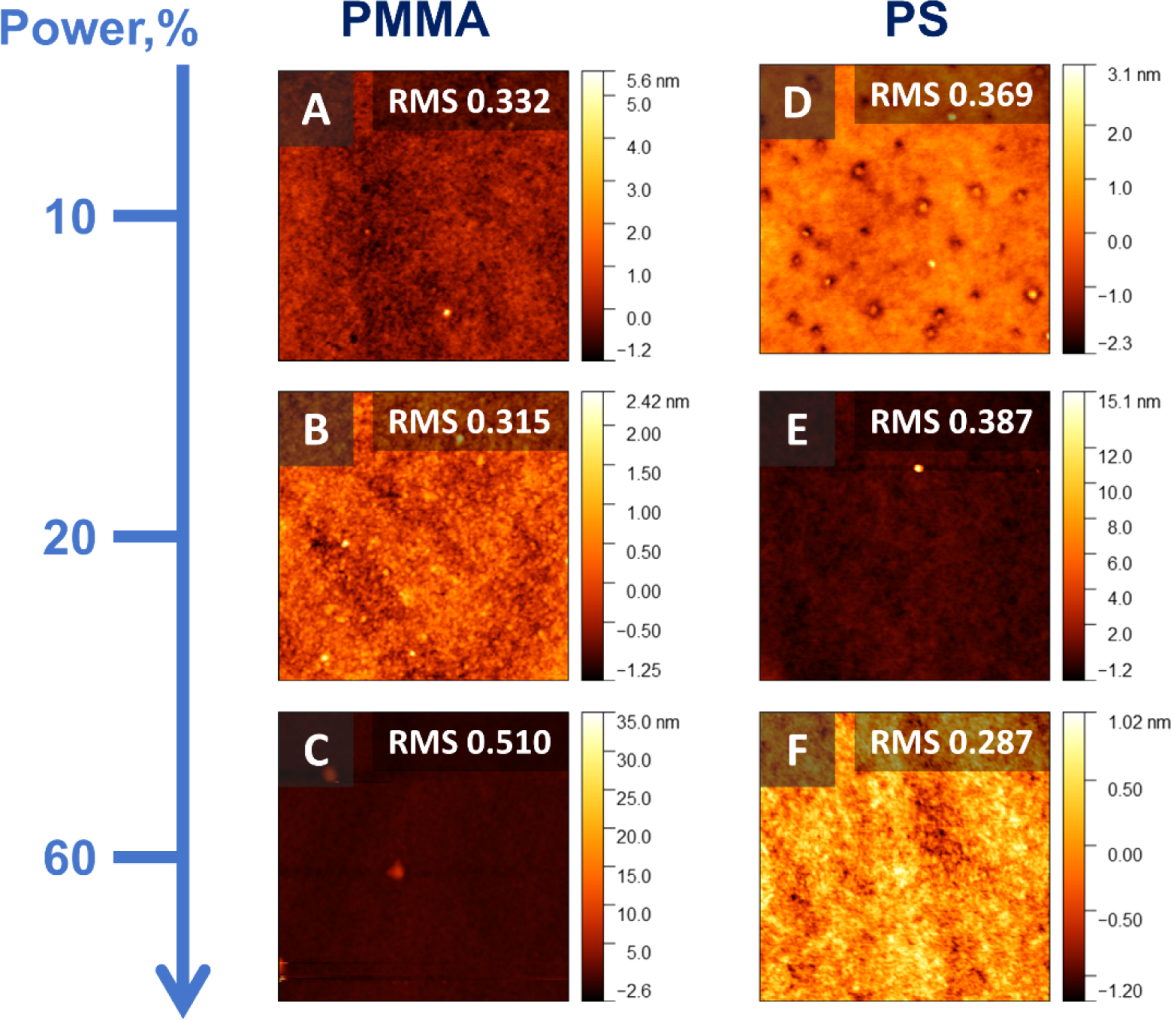
AFM micrographs (1 × 1 μm^2^) of plasma-treated PMMA (A–C) and PS (D–F) brushes after 1 min oxygen plasma treatment with associated ashing power and RMS roughness values are also shown in the insets.

The ellipsometry technique (Figure 5) was used to determine whether the brush degrades after UVO and plasma application, and indeed, a drop in thickness was observed with progressive treatment. The initial thicknesses for 6k PMMA brush and 10k PS brush were noted as 2.5 and 5 nm, respectively. The PS brush thickness is consistent with our previous study whereby transmission electron microscopy (TEM) analysis was used.^27^ The UVO treatment shows considerable degradation after 1 min for both PS and PMMA brushes, which is consistent with the AFM and CA data. Upon surface activation, the brush is immediately altered; however, compared to the overall brush thickness, the degree of degradation is negligible, i.e., < 0.5 nm degradation after 45 s UVO is observed for PMMA. Therefore, an activation time ∼45 s is optimal for PMMA based on CA, AFM, and ellipsometry analyses. Conversely, the degradation for PS appears to be more rapid ∼1.2 nm after 45 s and ∼1.5 for 60 s. However, the surface hydrophilicity of PS only increases upon 60 s treatment time (based on the CA results above).

**Figure 5:**
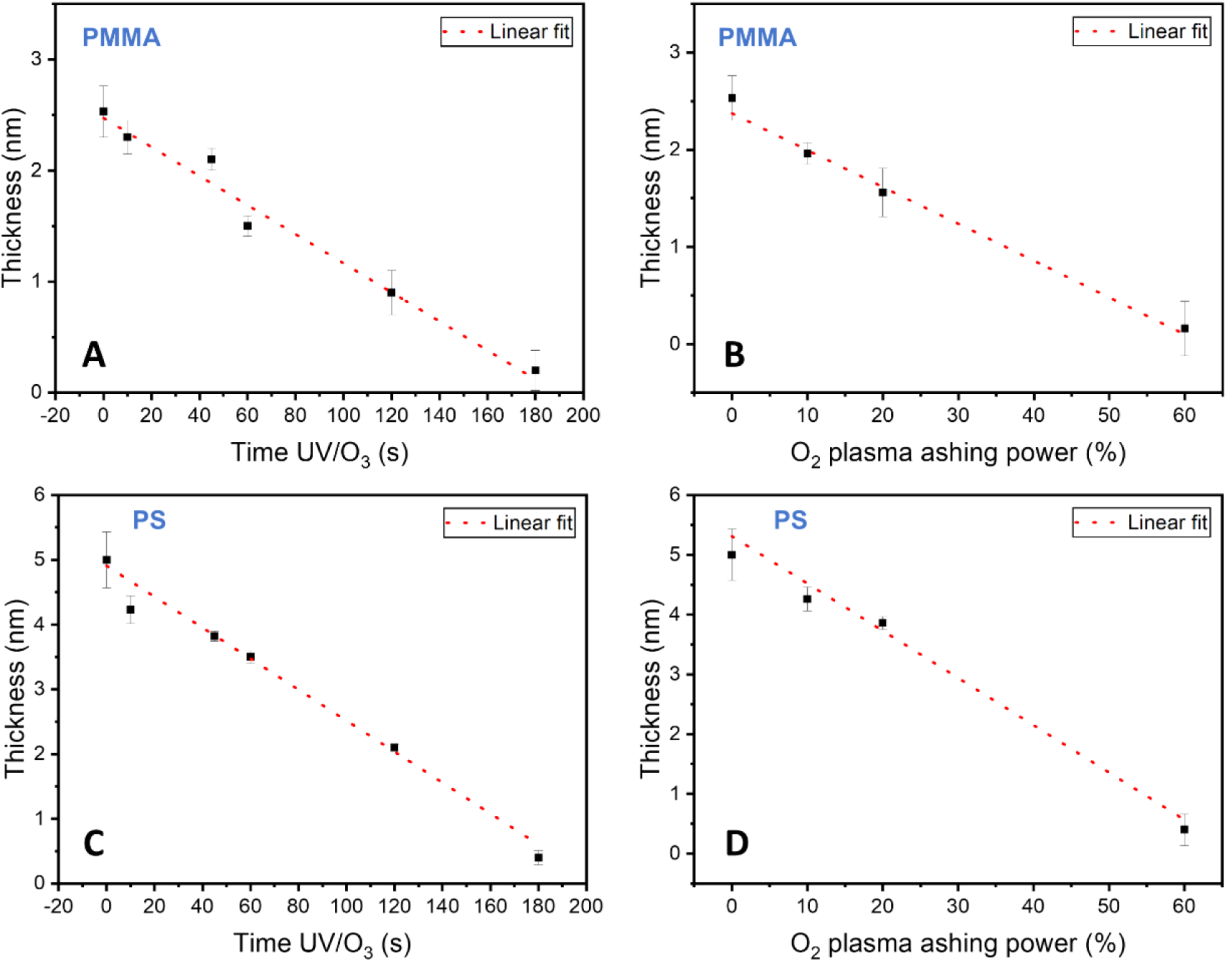
Ellipsometry thickness measurement as a function of UVO treatment time for PMMA (**A**) and PS (**C**) and as a function of oxygen plasma power treatment after 1 min for PMMA (**C**) and PS (**D**) respectively.

O_2_ plasma appears to be a more aggressive treatment as after 20% plasma treatment for 1 min approximately 1 nm is removed for both brushes. The 10% plasma is optimal for surface cleaning of PS and PMMA as <1 nm of material is removed. However, the 20% plasma treatment is essential for surface activation based on results from the CA analysis.

After 180s of UVO time, both brushes appear to be nearly fully degraded, which is also observed clearly from the AFM images in Figure 3. Similarly, 60% plasma treatment for 1 min results in the degradation of the brushes.

We anticipate that these findings can be used to aid the design of experiments for brush removal, especially in ASD with metal infiltration, whereby long UVO or plasma exposure would degrade the metal moiety. Furthermore, this shows that surprisingly short processing times are required for brush degradation.

### APTS Deposition on Polymer Brushes

Initial APTS deposition was optimized on PMMA homopolymer powder as detailed in the **Experimental section**. The chosen conditions were derived from the Antoine Equation, as shown in Figure S1 and are optimal for laboratory-based vapor deposition avoiding low pressures.^28^ The coverage on PMMA powder was estimated from the Ninhydrin test with a calibration plot shown in Figure S2A.

The relative coverage is stable after 6 to 10 h within the experimental error bound as seen from Figure S2B. Thus, the optimum annealing time was determined to be 6 h. Figure S2C demonstrates no considerable difference between FTIR spectra for 6 to 10 h deposition time, as the -NH_2_ stretching vibration at 3458 cm^–1^ is observed with similar intensities.

APTS was deposited atop PMMA and PS brushes according to the optimized process detailed above on 20% plasma-activated surfaces as well as a top a plasma-activated silicon substrate. The plasma-activated substrates were chosen as the CA analysis shows more hydrophilic character than UVO-activated substrates before substantial brush degradation occurs.

The coverage was accessed with CA, FTIR, and ToF-SIMS analysis. APTS stand-alone brush was formed atop a clean silicon wafer as shown in the AFM micrographs in Figure S3. It is important to note that the APTS deposition on nonactivated PMMA and PS brushes yields no significant change in CA from the pure PMMA and PS brush state, signifying that brush activation is essential (Figure S4A,C). A full APTS coverage is observed for PS and PMMA activated brushes, as seen from AFM and CA analyses (Figures 6 and S4B,D). The AFM analysis for the APTS-PS and APTS-PMMA indicates smooth films as well as observed from the RMS values as seen in Figure 6A-D. Furthermore, low CA hysteresis values confirm that smooth film surfaces are obtained (Figure 6E). The observed CAs are similar for APTS-coated silicon wafers as well as PS-APTS and PMMA-APTS modified brushes as observed from Figure 6. The two-sample *t*-test between the two sets of CAs (advancing and receding) for APTS and the APTS-polymer brush samples showed no significant differences (p≫0.05), verifying that APTS is indeed deposited atop the polymers.

**Figure 6:**
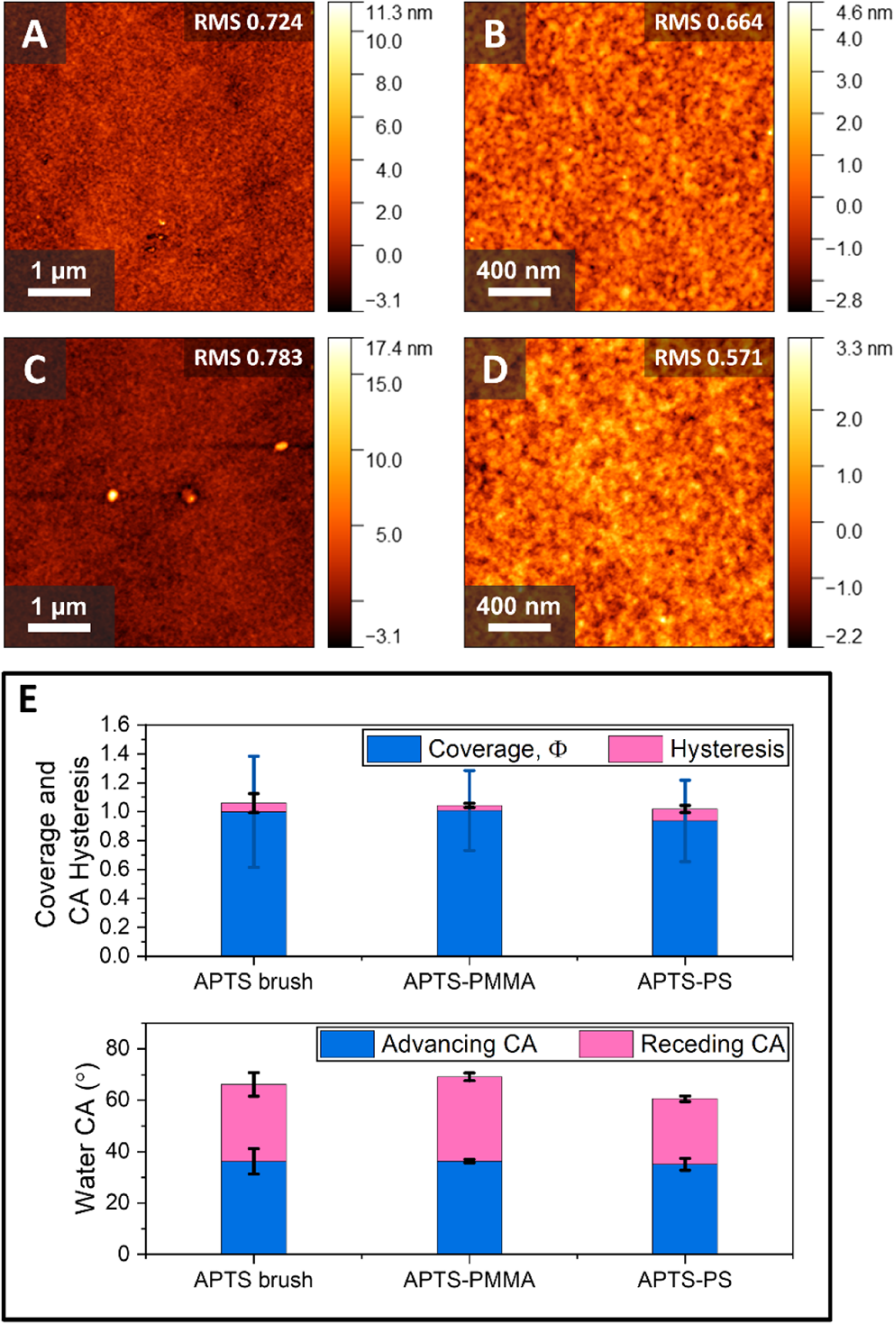
AFM micrographs of APTS deposited on: (**A**) and (**B**) 20% plasma-activated PMMA, and (**C**) and (**D**) 20% plasma-activated PS surfaces, with associated RMS roughness values. (**E**) CA analysis of the APTS brush atop silicon and APTS deposited on 20% plasma-activated PMMA and PS surfaces. The associated coverage and hysteresis are displayed. The coverage is compared to an APTS brush for which the coverage is set as 1.

The water CA measurement remained unchanged for all brush samples (PMMA, PS, PMMA-APTS, and PS-APTS) for a period of up to 2 weeks from the date of sample preparation, indicating sample stability and uniformity despite storage under ambient lab conditions (pressure ∼1 bar, humidity ∼ variable 40–70%). Similarly, the physical sample appearance under AFM or SEM remained unchanged up to 2 weeks after sample preparation, but longer periods of storage were not tested. The contact angle measurement is more reliable than microscopic techniques for testing chemical stability and surface purity; thus, we can deduce that dust and physiosorbed contaminants begin to accumulate on the surface after 2 weeks. We advise that care should be taken to avoid surface contaminants, and thus storage under vacuum could be beneficial.

Figure S5 SEM micrographs show the visual brush uniformity for PS, PMMA, and APTS brushes. The δ­(NH_2_) mode at 1568 cm^–1^ (PMMA) and 1571 cm^–1^ (PS) observed in the grazing angle FTIR spectra confirms the presence of APTS on the brushes as portrayed in Figure S6.^29,30^ Furthermore, other bands confirm the presence of the APTS moiety, as detailed in Table S1.

The samples were analyzed by ToF-SIMS to ultimately verify whether the APTS is uniformly bound to the surface. The fingerprint spectra (positive ion, *m*/*z* = 3–300) for the analyzed materials are shown in Figure S7. The PCA analysis was also conducted on selected sample images as displayed in Section S2 and Figures S8–S14, showing macroscale ion distribution on the samples. Figure S15 shows 2D surface maps of specific ions of interest for the PMMA and PS brush samples before and postplasma treatment and for the corresponding APTS-polymer brush samples. The ions attributed to polymer brushes are compared to the APTS molecular ion (C_9_H_23_NO_3_Si^+^), and we can visually observe the change after APTS grafting onto the surfaces. The APTS terminal amine group can form a hydrogen bond with a hydroxyl group of the activated brush surfaces; thus, the APTS molecular ion was detectable.^14,31^ Furthermore, we chose the APTS molecular ion, as it is more easily distinguishable via ToF-SIMS from the brush surfaces compared to other ions such as NH_4_^+^ or Si^+^ or various organic chains that could be attributed to contaminants or the polymer brushes themselves. The CH_3_O^+^ and C_2_H_4_O^+^ ions were selected for PMMA,^32^ and C_6_H_5_^+^, C_7_H_7_^+^ for PS.^33^ The APTS ion was designated by the color red, and the overlay images evidently show a tendency toward red for PMMA-APTS samples. For the PS samples, the coverage appears patchier based on the overlay images. However, the intensity of the APTS ion is higher for both APTS-polymer brush samples versus the corresponding brush substrate samples. Figure S16 further compares the 2D maps of the APTS fragments (CH_4_N^+^, C_2_H_6_N^+^ and C_3_H_8_N^+^)^34^ similarly to Figure S15. It is evident that the plasma-activated samples show overlapping signals in the regions corresponding to the ions above, and thus, small fragments are not reliable for elucidating APTS binding in this case. Nevertheless, this visual analysis should only be used qualitatively as matrix effects^35,36^ were not eliminated.

PCA analysis was performed to elucidate whether the binding of APTS to the polymer substrates was effective versus plasma-activated samples and pristine brush samples. PCA helps us to understand differences between groups of samples depending on the inputs (which in our case are the identified ions). 115 positive ions were chosen as listed in Table S1. There is an overlap between ions attributed to the surfaces under investigation; thus, it is more useful to look at scores and loadings derived from PCA directly. In simple terms, the PC1 component represents the most variation in the data, while PC2 represents the second most variation in the data. By comparing variations in the signals of these 115 ions (specifically the peak areas) for each sample individually, we can observe that samples representing unique conditions or scenarios are grouped together by these variations in the plot of PC1 vs PC2 (Figure 7). There are 4 different scenarios that all occupy different quadrants of the scores on PC2 versus PC1 plots for both PMMA and PS as expected. It is noted that the bounding ellipses are representative of 68% confidence for visual purposes as 95% confidence with only 3 samples would produce a much larger spread of possible PC1 vs PC2 areas. However, this simple analysis combined with previous data such as FTIR and contact angle demonstrates that all treatments, i.e., brush deposition, oxygen plasma treatment, and APTS binding, were successful.

**Figure 7:**
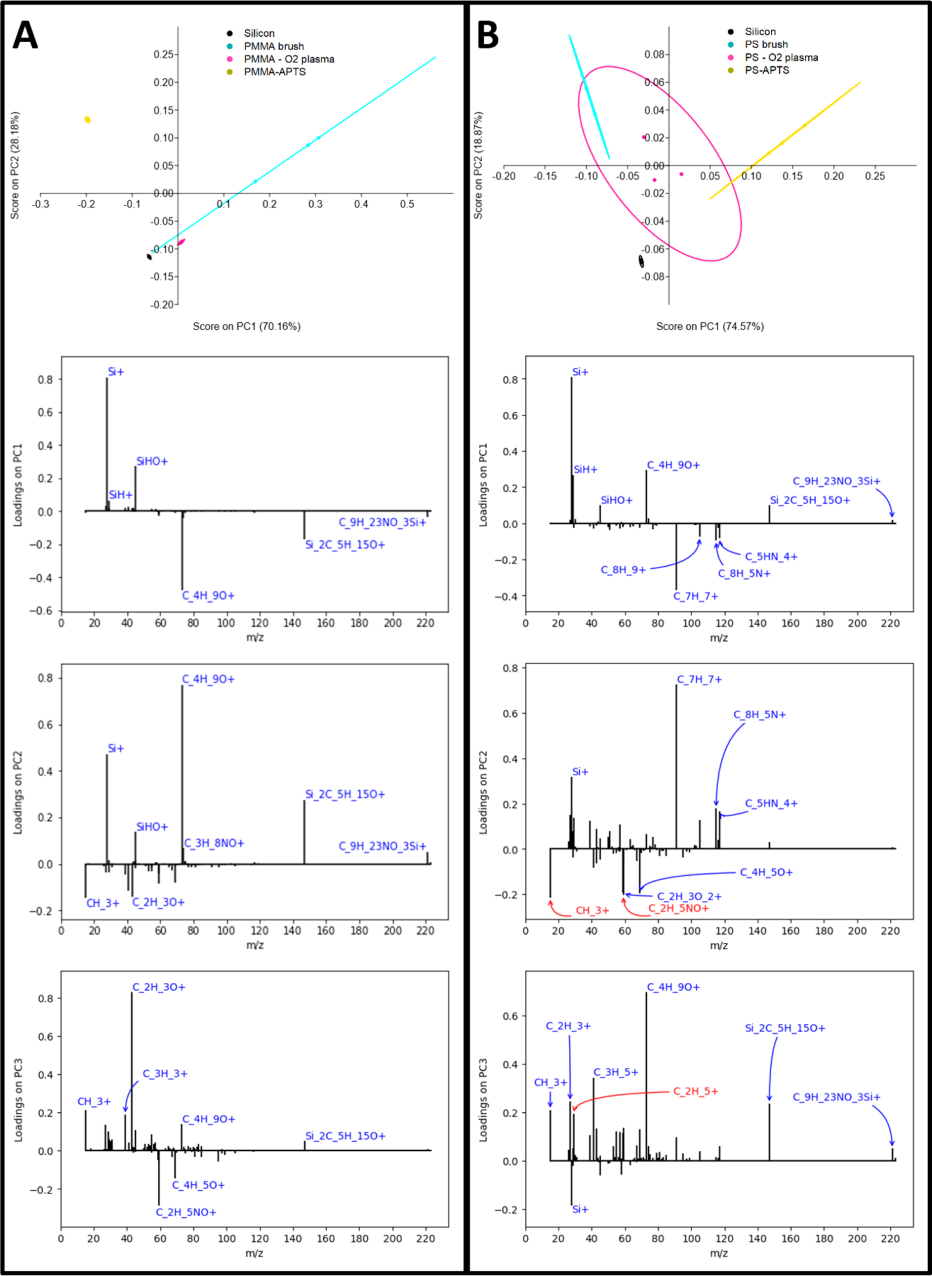
PCA analyses for (A) PMMA and (**B**) PS brushes displaying scores and loadings for the substrate (Si), brush, plasma-activated brush, and APTS-polymer brush. The ellipses in the score plots indicate 1 sample standard deviation.

The scores on the PCA plots are substantially different between APTS-PS and APTS-PMMA compared to the corresponding polymer brush, as shown in Figure 7. APTS-polymer brush samples represent negative scores in our case on both PC1 and PC2, indicating successful deposition. The loadings for individual ions show which ions are responsible for the most variation in each successive PCA component; i.e., PC1, PC2, and PC3 are shown in Figure 7. The ions associated with loadings of interest are labeled on the respective loading plots.

In simple terms, PC1 or the most variation within data is attributed to silicon ion content and oxygenated polymeric ions as expected i.e., silicon wafer vs brushes. The negative loadings indicate ions responsible for the APTS groups on brushes (such as C_9_H_23_NO_3_Si^+^ which is the APTS molecular ion among others); the effect is more evident in the loadings on PC2. Second most variation (PC2) for PS sample group is due to polymeric chains, for example, C_7_H_7_^+^ ion can be associated with PS. For the PMMA sample group, several small positive loadings on PC2 represent nitrogen- or silicon-containing groups, which can be attributed to the APTS moiety; this effect is more prominent for the PMMA-APTS samples. Finally, the PC3 loading plot shows positive loadings associated with APTS becoming more prominent for the PS-APTS sample. Si_2_C_5_H_15_O^+^ is identified as a contaminant often associated with PDMS or other silanes^37^ thus is not conclusive of APTS binding. Overall, APTS binding is only obvious from the PC2 and PC3 components; i.e., the C_9_H_23_NO_3_Si^+^ positive loading is more prominent than for PC1. This is expected as the sample groups are distinctly varied (silicon wafers, brush samples, and APTS-polymer brush); thus, most variation would be due to bulk component differences.

Further analyses show a uniform surface distribution, as indicated in Section S2, where PCA 2D plots are shown per select samples (Figures S8–S15). However, for the purpose of this study, it is more useful to compare all APTS samples versus each experimental stage. Thus, ToF-SIMS in this case supports the presence of APTS on the surface.

For a full quantitative analysis via ToF-SIMS a calibration standard should be used per polymer brush type. Thus, a future study would include a more detailed ToF-SIMS method to account for matrix effects as well as provide a calibration technique. The thickness of the APTS layers cannot be deduced from ToF-SIMS and AFM analyses alone, so further study should be conducted to investigate layer depth in 3D. Nevertheless, the brushes can act as substrates for other chemisorbed layers themselves, as shown by this study.

## Conclusion

We demonstrated a degradation and an activation scheme for two different polymer brushes, namely, PMMA and PS. The brushes can be activated, i.e., the surface hydrophilicity can be increased, by either UVO or plasma for surface functionalization with moieties such as APTS. The optimal treatment for brush activation is proposed to be oxygen plasma treatment versus UVO as lower sample degradation coupled with adequate hydrophilicity increase is observed. Only 1 min of processing time is required for oxygen plasma at low powers, while 1 min of UVO processing time leads to surface activation as well as considerable surface degradation (ca. > 1 nm). Furthermore, APTS treatment of the activated brushes yields APTS-PMMA and APTS-PS stacks as verified by FTIR, CA, AFM, and ToF-SIMS analyses. The possibility of brush alteration postdeposition should open new avenues for research and applications, such as versatile ASD and regional selectivity for other chemical binding, such as proteins. Where the surface chemistry of the substrate only allows specific brushes to be deposited, for example, in medical applications where low toxicity is essential or for other reasons, our strategy of brush activation and stacking offers an advantage. Thus, we believe that this work will be useful for extending knowledge in various areas ranging from materials science to biological fields.

## Supplementary Material



## Notes

APTS deposition optimization via Ninhydrin test and additional AFM, SEM, FTIR, CA and ToF-SIMS data (PDF)
