# Supplementary material for: Enhanced Binding of (3-Aminopropyl)triethoxysilane to Polymer Brush-Coated Surfaces by Controlled Activation: Degradation, Activation, and Functionalization
Source: ACS Omega. 2025 Aug 29;10(36):41926–37. doi: 10.1021/acsomega.5c06525 (PMC12444495; doi:10.1021/acsomega.5c06525)
Supplement: Supplementary file 1 [file ao5c06525_si_001.pdf]

Supporting Information

Enhanced binding of (3-aminopropyl)triethoxysilane to polymer brush coated surfaces by controlled activation: degradation, activation and functionalization.

Nadezda Prochukhan,[a,b,*] Arantxa Davó-Quiñonero,[a,c] Alberto Alvarez-Fernandez,[a] Pravind Yadav,[a] Sergey Beloshapkin,[d] Abbie Jane Keegan,[a] Ross Lundy,[a] Michael A. Morris[a,b,*]

[a]*School of Chemistry, CRANN and AMBER Research Centres, Trinity College Dublin, College Green, Dublin 2, Ireland.*

[b]*BiOrbic — Bioeconomy SFI Research Centre, University College Dublin, Belfield, Dublin 4, Ireland*

[c]*University of Alicante, Carretera San Vicente del Raspeig s/n, Alicante, Spain 03690*

[d]*Department of Chemical Sciences, Bernal Institute, University of Limerick, V94 T9PX Limerick, Ireland*

Section S1 – APTS deposition optimization and supplementary analyses

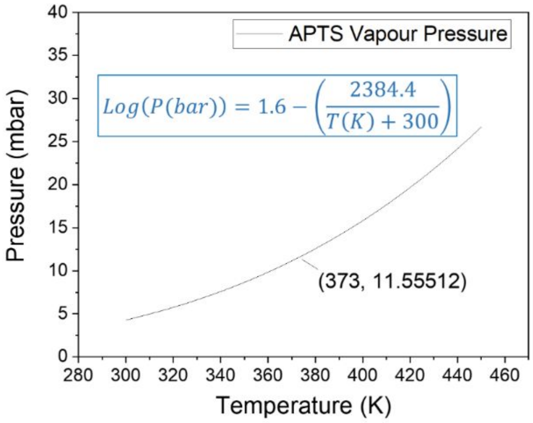

Figure S1. APTS Antoine Equation parameters[1] used to design deposition conditions (300-390 K range).

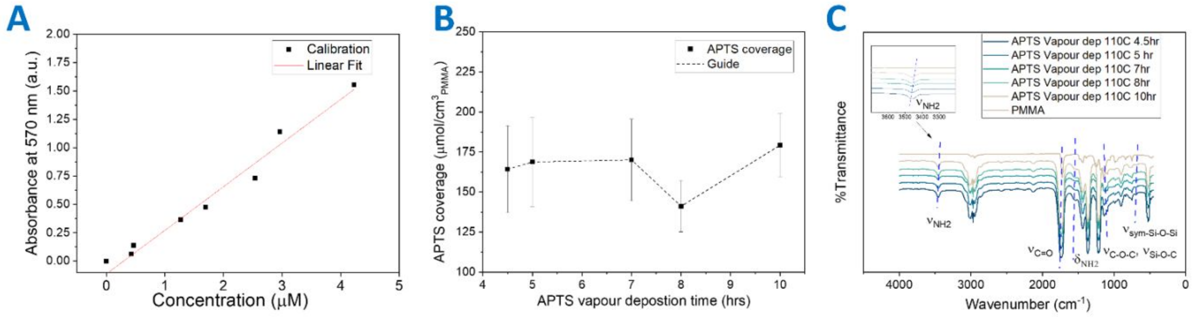

Figure S2. Ninhydrin optimisation. **A.** UV-Vis calibration curve for APTS using Ninhydrin test. **B.** Coverage on PMMA powder (Altuglas, assuming 0.7 g cm^{-3} density) after APTS vapour deposition estimated from the Ninhydrin test. **C.** FTIR spectra of PMMA and APTS-functionalised PMMA powder.

The Ninhydrin test was used as a guide to determine optimal conditions for APTS deposition. PMMA powder was used as a test substrate due to the ease of measuring the Ninhydrin test product (purple colour) with UV-Vis spectroscopy. APTS was also reacted with Nynhydrin to produce the blue purple adduct for calibration. Thus, peak absorbance at 570 nm was calibrated versus APTS concentration in micromolar. Subsequently, the test was conducted on ground PMMA powder of known mass. The density of PMMA as reported by Altuglas is 0.7 g cm^{-3},

thus we can obtain the APTS coverage over PMMA volume as shown in **Figure S2 B**. The graph is purely indicative of the coverage as grinding powder can improve bulk density which can be between 0.7 g cm^{-3} and the PMMA bulk density of 1.18 g cm^{-3}. **Figure S2 B** demonstrates peak coverage doesn't change considerably after 4 – 7 hrs of deposition which is predicted to be limiting for this sample. This is also true as the powder sample was stored in a vial during deposition and some faces of the powder particles could be exposed less to the APTS vapour. Thus, we can postulate that sufficient time was given as all possible orientations of the power were coated with APTS. This preliminary information is required to design subsequent experiments on polymer brush thin films as detailed in the manuscript. The FTIR spectrum in **Figure S2 C** simply demonstrates the increase in the transmittance intensity of the amine stretching mode indicative of APTS deposition.

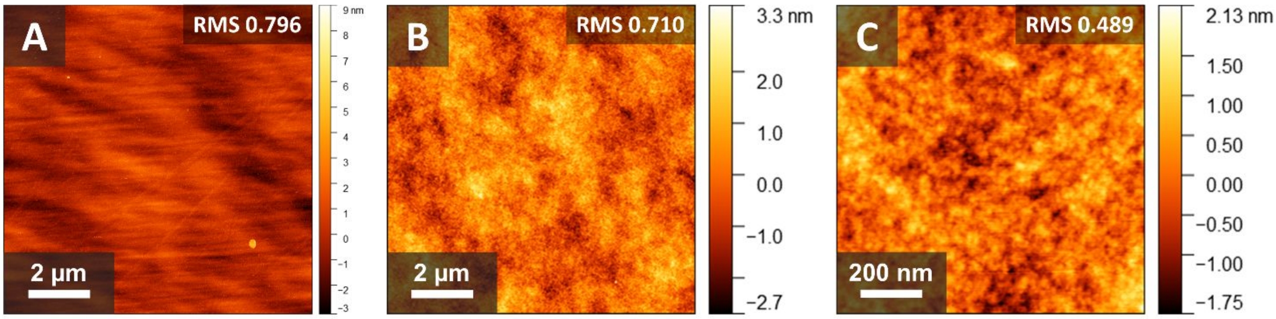

Figure S3. AFM micrographs of **A.** clean Si wafer, **B.** and **C.** APTS brush with associated RMS roughness.

<u>Notes on choice of polymers:</u>

The grafting density for APTS was not explored as it is predicted to form monolayers on the surface, however, we define surface coverage by contact angle analysis which was observed to be ~100%.

The polymer concentrations are different as optimised and explained in our previous studies.[2,3] The use of different concentrations with polymers of similar sizes is explained by many factors such as binding affinity, polymer mobility, polymer free volume, steric effects that can prevent effective binding, solubility of polymer in solvent etc. These studies have also been references in the text.

The thickness is also variable due to different chain lengths of polymers used i.e., PS molecular weight used was 10kg/mol while PMMA was 6 kg/mol.

Simply, using the polymer physical models such as modelling polymer chain length given expected conditions we can calculate the expected chain length.

Firstly, the degree of polymerization is simply the weight of the chain divided by the monomer mass:

Degree of polymerization of PMMA = $N_{PMMA} = \frac{6000}{100.117} = 59.9 \sim 60$

And $N_{PS} = \frac{10000}{104.152} \sim 96$

The formula for chain length is $R = bN^{\frac{1}{2}}$, where b is the Kuhn monomer length which is tabulated[4] as $b_{PMMA} = 1.7\,nm$ and $b_{PS} = 1.8\,nm$. Thus, the ideal chain length is $R_{PMMA} = 13\,nm$ and $R_{PS} = 17\,nm$. Thus, we can see even theoretically the expected chain lengths are different and thus when the polymer brush is formed we expected the thickness of the layers to be different too.

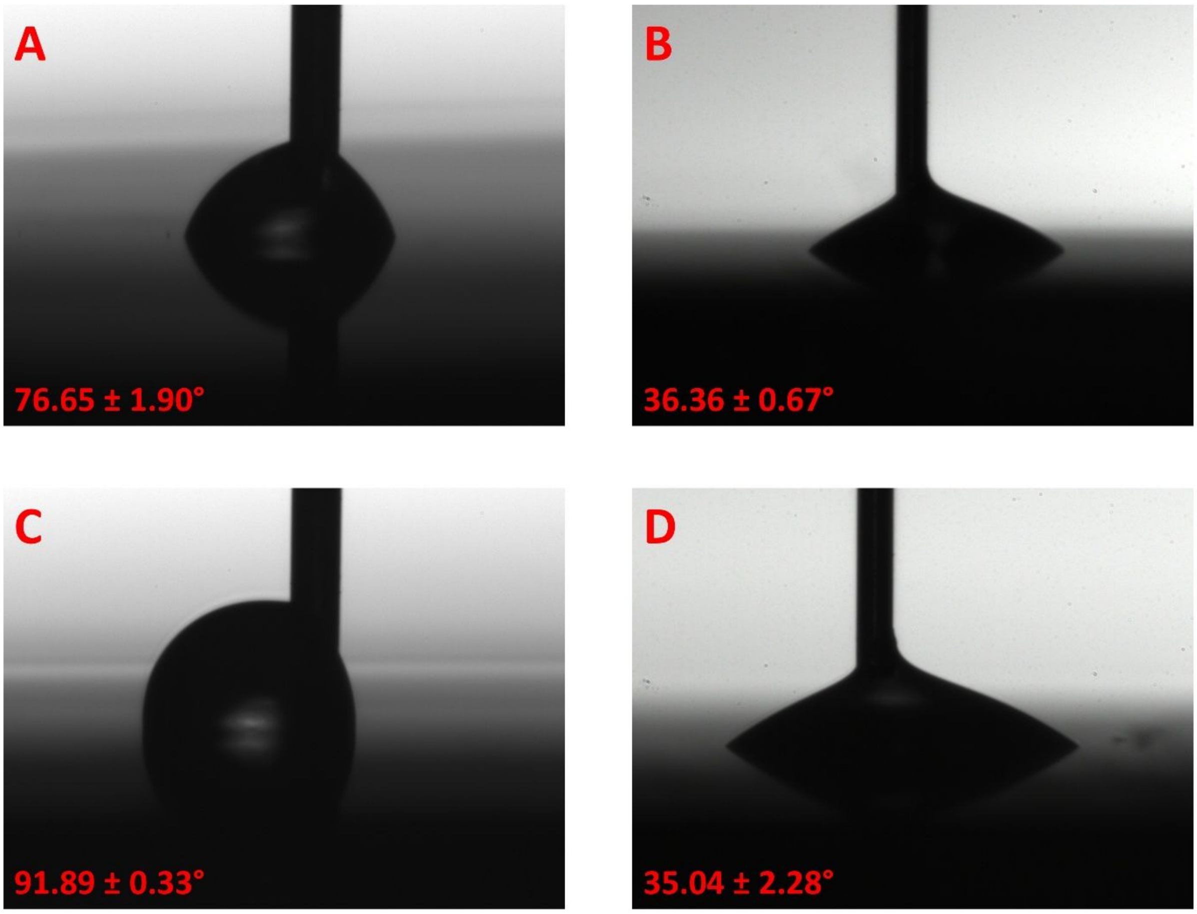

Figure S4. Contact angle (CA) analysis of the samples post APTS deposition atop of **A.** PMMA brush, **B.** activated PMMA brush (20% plasma), **C.** PS brush and **D.** activated PS brush (20 % plasma). The APTS successfully coats only plasma activated brushes.

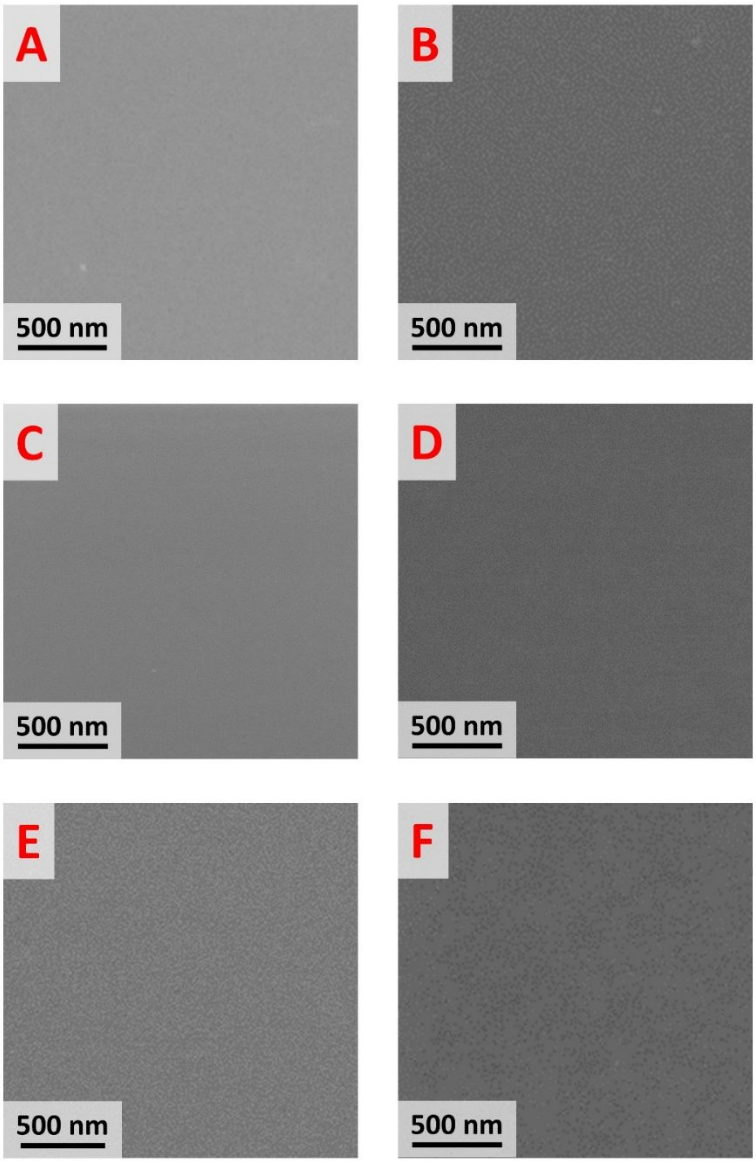

Figure S5. SEM micrographs of **A.** Silicon wafer, **B.** APTS on Si, **C.** PMMA brush, **D.** PMMA-APTS, **E.** PS brush and **F.** PS-APTS.

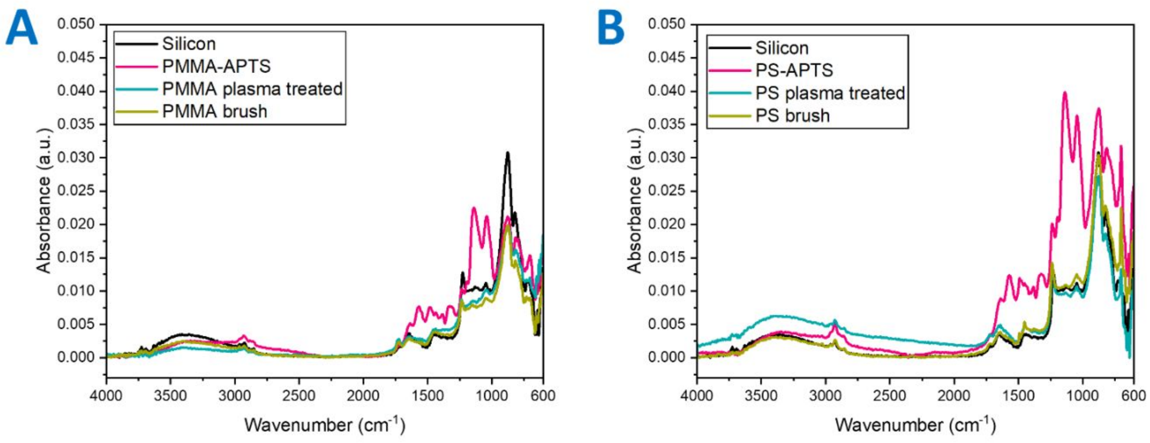

Figure S6. FTIR analysis of **A.** PMMA and **B.** PS brushes versus the silicon substrate, after plasma treatment (20%, 1 min) and after APTS deposition.

Table S1. Characteristic FTIR peaks identified for APTS deposited atop PMMA and PS brushes.

**Wavenumber, cm^{-1}**		**Assignment**	**Reference**
**APTS-PMMA**	**APTS-PS**		
1568	1571	NH_2 deformation	[5,6]
1385	1384	Symmetric stretch C-N	[6,7]
1305 – 1340 (broad)		O-H in plane bending, Si-CH_2 wag	[6]
1140	1138	Asymmetric stretch (Si-O-C)	[5,6]
1039	1044	Symmetric stretch (C-O)	[5,7]

Section S2 – supplementary ToF-SIMS data

Table S2. Characteristic ToF-SIMS peaks selected for principal component analysis.

CH_3^+	$SiCH_4^+$	$SiC_2H_6^+$	$C_5H_{10}^+$	$C_2H_3N_3O^+$
OH^+	$C_2H_4O^+$	$C_3H_6O^+$	$C_4H_7O^+$	$C_5H_9O^+$
NH_3^+	$C_2H_6N^+$	$C_3H_8N^+$	$SiC_3H_8^+$	$SiC_4H_{10}^+$
NH_4^+	$SiHO^+$	$C_4H_{10}^+$	$C_4H_8O^+$	$C_2H_3N_2O_2^+$
$C_2H_2^+$	$SiCH_5^+$	$C_2H_3O_2^+$	$C_4H_9O^+$	$C_4H_9NO^+$
$C_2H_3^+$	$C_2H_5O^+$	$C_2H_5NO^+$	$C_3H_8NO^+$	SiC_5H^+
Si^+	CH_4NO^+	$C_3H_7O^+$	$C_6H_3^+$	C_6HO^+
CO^+	SiH_3O^+	$C_2H_4O_2^+$	$C_3H_7O_2^+$	$C_7H_7^+$
SiH^+	$CH_3O_2^+$	$CH_6N_3^+$	$C_6H_4^+$	$C_7H_{11}^+$
CHO^+	$C_2H_7O^+$	$SiCH_7N^+$	$SiC_2H_{10}N^+$	$C_5H_7NO^+$
CH_3N^+	$C_4H_2^+$	NO_3^+	SiC_4H^+	$C_7H_{13}^+$
$C_2H_5^+$	$C_4H_3^+$	$C_2H_4OF^+$	$C_6H_5^+$	$C_5H_9NO^+$
SiH_2^+	$C_4H_4^+$	SiH_6NO^+	$Si_2H_6O^+$	$SiC_3H_8NO^+$
CH_4N^+	C_3HO^+	$C_4H_3N^+$	$C_6H_6^+$	$SiC_6H_3^+$
SiH_3^+	$C_4H_5^+$	C_3NO^+	$C_6H_7^+$	$C_8H_9^+$
CH_3O^+	CH_3ONa^+	$C_4H_4N^+$	$C_6H_8^+$	$C_8H_5N^+$
CH_6N^+	$C_4H_6^+$	SiC_2HN^+	$C_5H_5O^+$	$C_6N_2O^+$
$C_3H_3^+$	$C_3H_3O^+$	$C_5H_7^+$	$C_6H_9^+$	$C_9H_8^+$
$C_3H_5^+$	$C_4H_7^+$	$C_2H_2N_3^+$	$C_5H_6O^+$	$C_5HN_4^+$
$C_2H_3O^+$	$C_3H_4O^+$	$C_5H_8^+$	$C_6H_{10}^+$	$SiCH_3N_2O_3^+$
$C_3H_7^+$	$C_4H_8^+$	$C_4H_5O^+$	$C_4H_3O_2^+$	$Si_2C_5H_{15}O^+$
SiO^+	$C_3H_5O^+$	$C_5H_9^+$	$C_5H_7O^+$	$C_9H_{23}NO_3Si^+$
SiH_2N^+	$C_4H_9^+$	$C_4H_6O^+$	$C_6H_{11}^+$	$C_{16}H_{15}O^+$

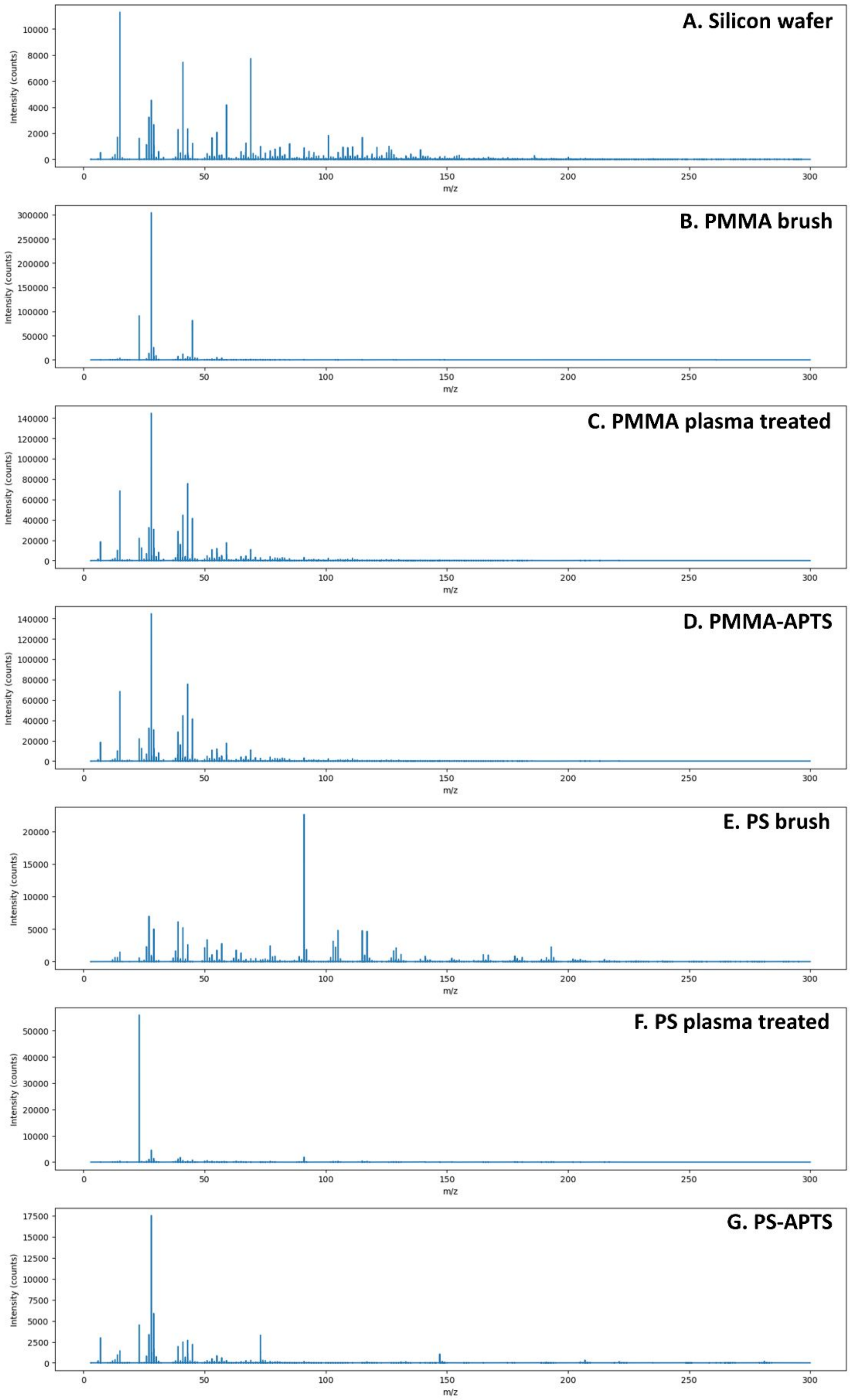

Figure S7. ToF-SIMS (positive ion) spectra of selected samples. **A.** Silicon, **B.** PMMA, **C.** PMMA-plasma treated, **D.** PMMA-APTS, **E.** PS, **F.** PS-plasma treated and **G.** PS-APTS.

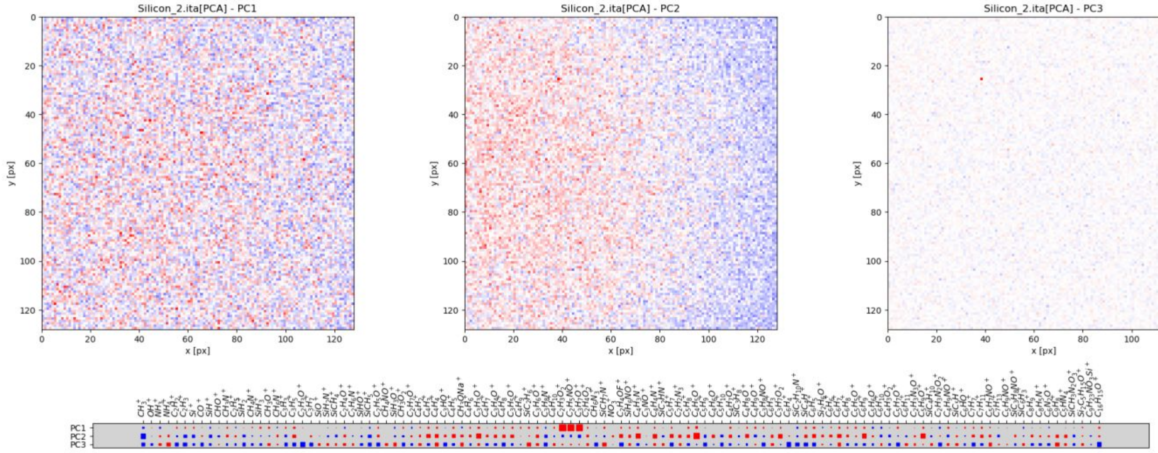

Figure S8. PCA on the silicon substrate showing PC1, PC2, PC3 and the loadings of the selected 115 ions. Red indicates positive loadings and blue indicates negative loadings.

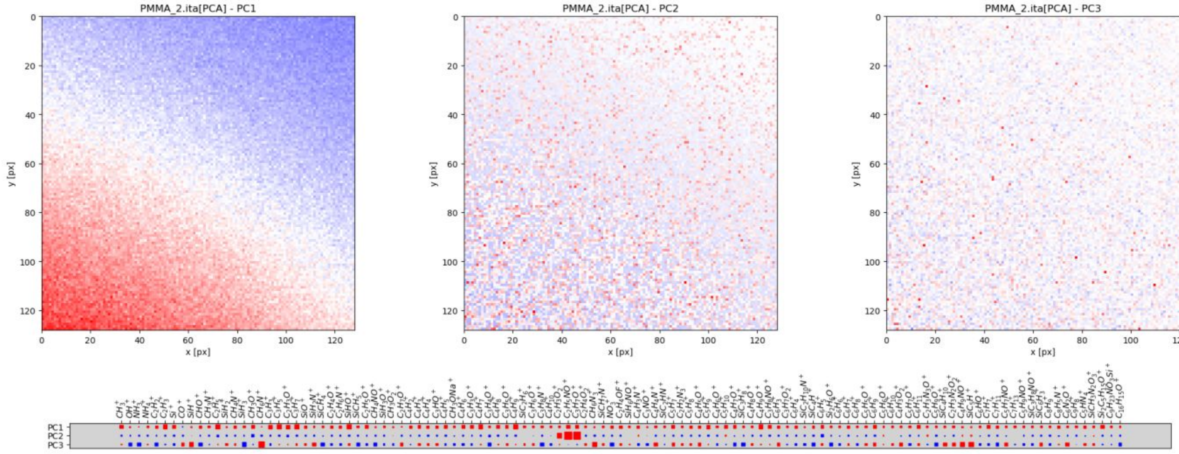

Figure S9. PCA on the PMMA brush showing PC1, PC2, PC3 and the loadings of the selected 115 ions. Red indicates positive loadings and blue indicates negative loadings.

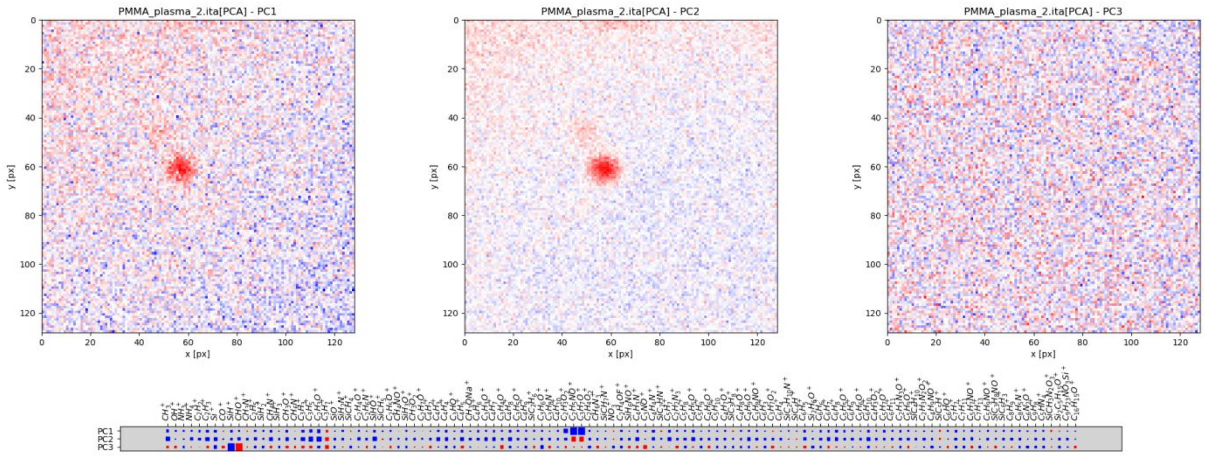

Figure S10. PCA on the PMMA brush after plasma treatment showing PC1, PC2, PC3 and the loadings of the selected 115 ions. Red indicates positive loadings and blue indicates negative loadings.

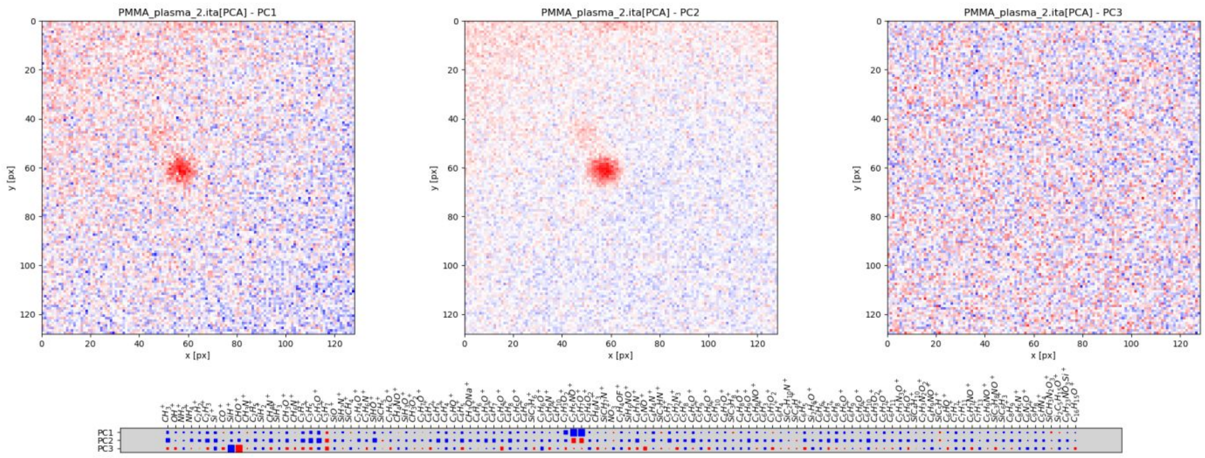

Figure S11. PCA on the PMMA-APTS showing PC1, PC2, PC3 and the loadings of the selected 115 ions. Red indicates positive loadings and blue indicates negative loadings.

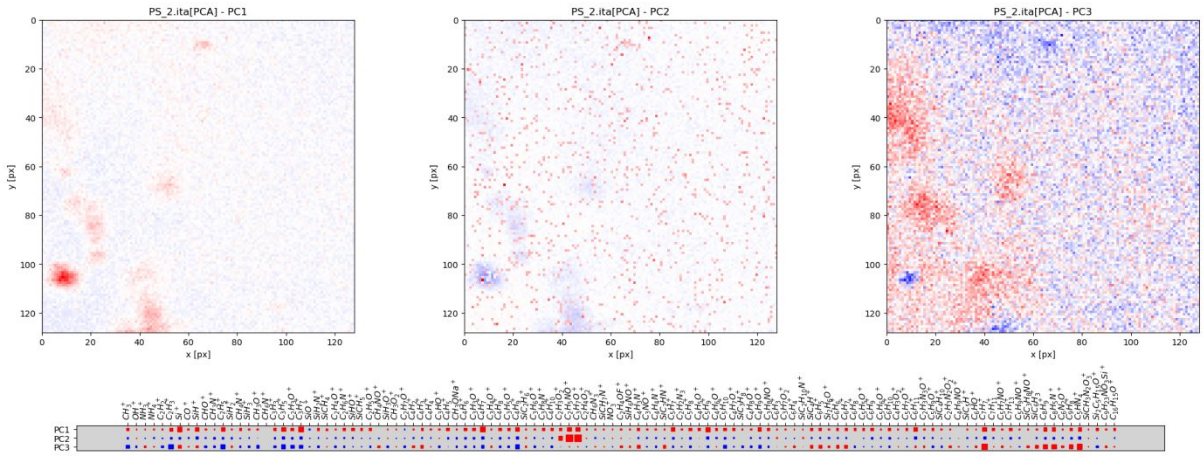

Figure S12. PCA on the PS brush showing PC1, PC2, PC3 and the loadings of the selected 115 ions. Red indicates positive loadings and blue indicates negative loadings.

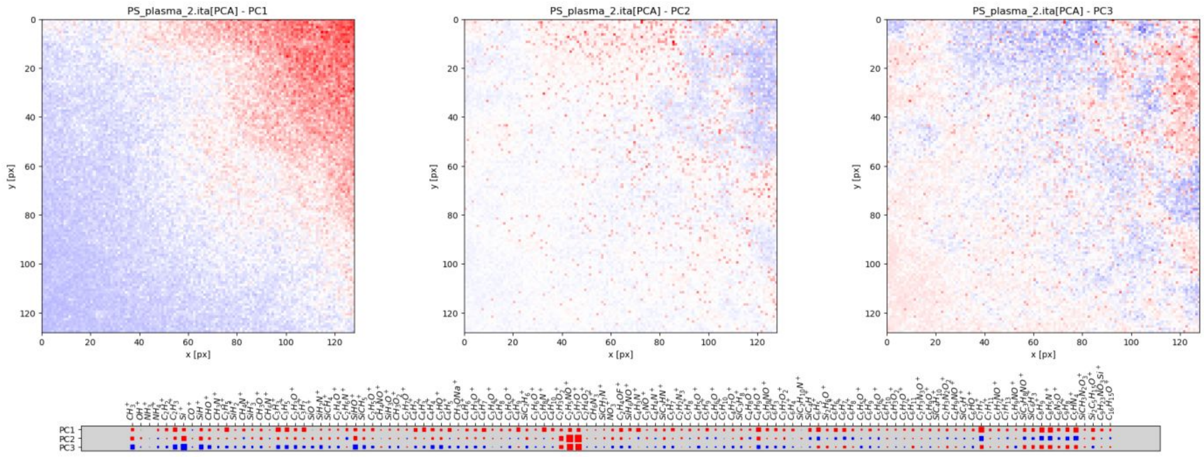

Figure S13. PCA on the PS brush after plasma treatment showing PC1, PC2, PC3 and the loadings of the selected 115 ions. Red indicates positive loadings and blue indicates negative loadings.

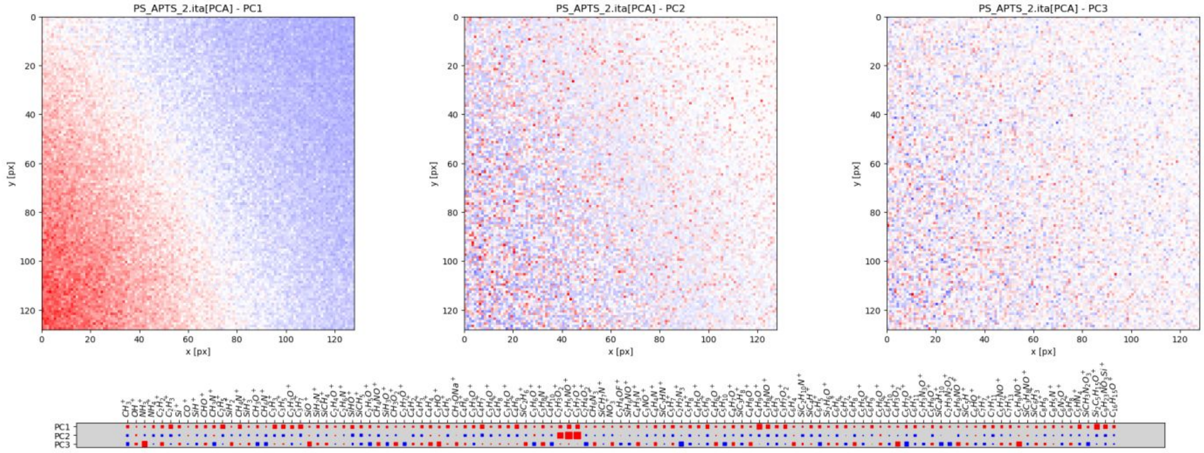

Figure S14. PCA on the PS-APTS showing PC1, PC2, PC3 and the loadings of the selected 115 ions. Red indicates positive loadings and blue indicates negative loadings.

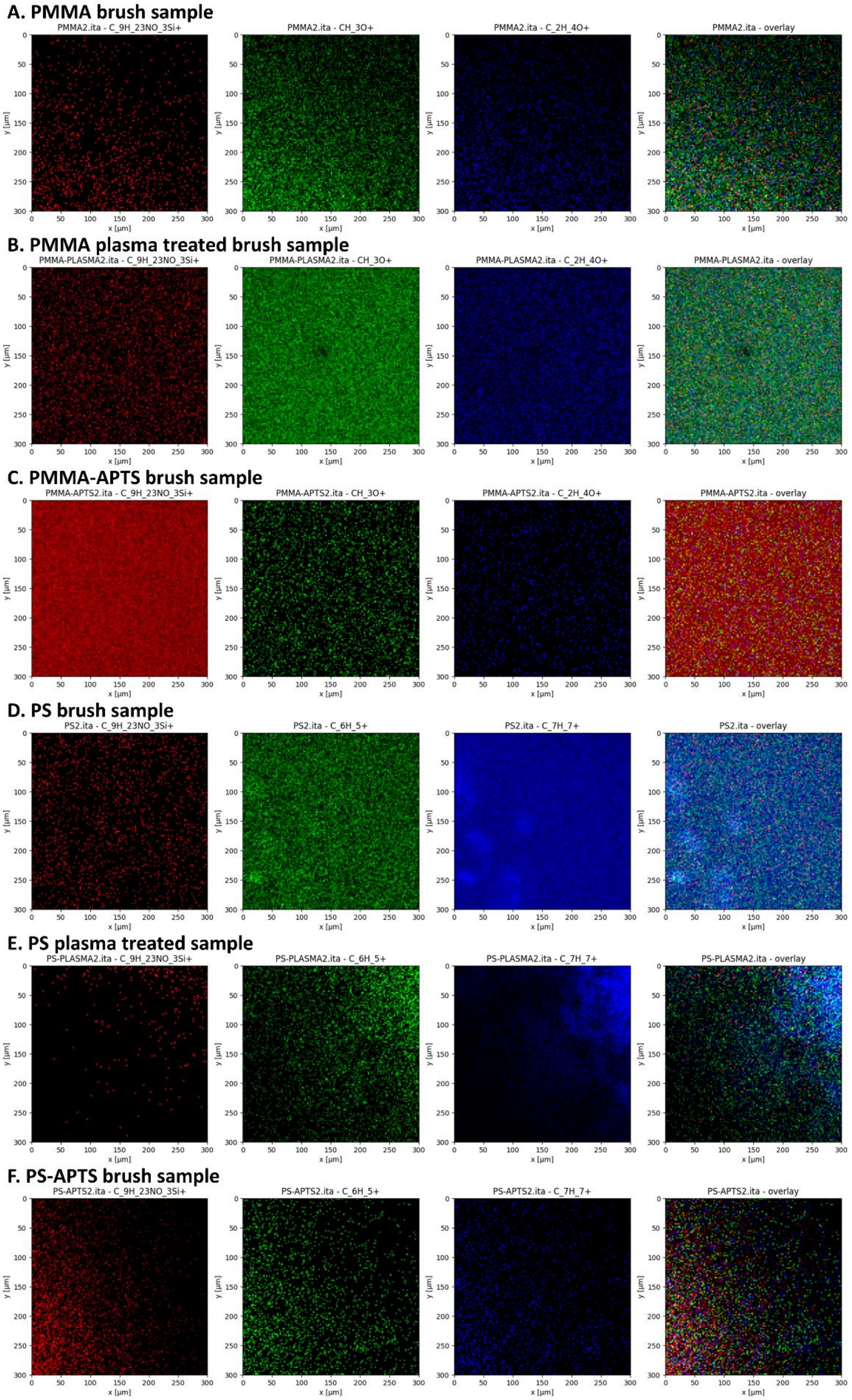

Figure S15. ToF-SIMS (positive ion) 2D maps of selected ions and samples with corresponding ion map overlays. **A.** PMMA, **B.** PMMA-plasma treated, **C.** PMMA-APTS, **D.** PS, **E.** PS-plasma treated and **F.** PS-APTS.

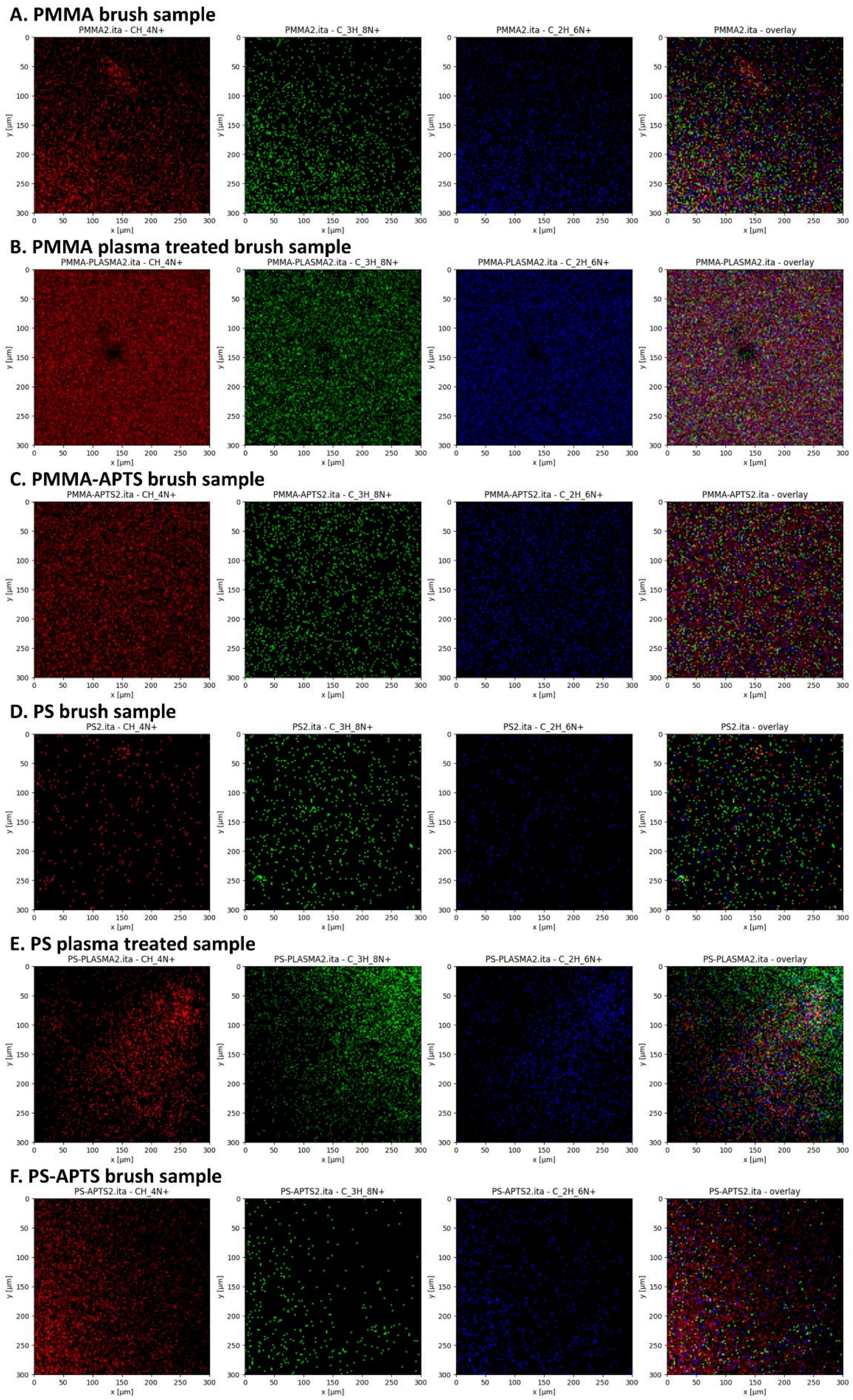

Figure S16. ToF-SIMS (positive ion) 2D maps of selected APTS fragment ions and samples with corresponding ion map overlays. **A.** PMMA, **B.** PMMA-plasma treated, **C.** PMMA-APTS, **D.** PS, **E.** PS-plasma treated and **F.** PS-APTS.

References

(1) Gueye, M.; Gries, T.; Noël, C.; Migot-Choux, S.; Bulou, S.; Lecoq, E.; Choquet, P.; Kutasi, K.; Belmonte, T. Interaction of (3-Aminopropyl)Triethoxysilane with Pulsed Ar–O2 Afterglow: Application to Nanoparticles Synthesis. *Plasma Chem. Plasma Process.* **2016**, *36* (4), 1031–1050. https://doi.org/10.1007/s11090-016-9708-3.

(2) Lundy, R.; Yadav, P.; Selkirk, A.; Mullen, E.; Ghoshal, T.; Cummins, C.; Morris, M. A. Optimizing Polymer Brush Coverage to Develop Highly Coherent Sub-5 Nm Oxide Films by Ion Inclusion. *Chem. Mater.* **2019**, *31* (22), 9338–9345. https://doi.org/10.1021/acs.chemmater.9b02856.

(3) Lundy, R.; Yadav, P.; Prochukhan, N.; Giraud, E. C.; O'Mahony, T. F.; Selkirk, A.; Mullen, E.; Conway, J.; Turner, M.; Daniels, S.; Mani-Gonzalez, P. G.; Snelgrove, M.; Bogan, J.; McFeely, C.; O'Connor, R.; McGlynn, E.; Hughes, G.; Cummins, C.; Morris, M. A. Precise Definition of a "Monolayer Point" in Polymer Brush Films for Fabricating Highly Coherent TiO2Thin Films by Vapor-Phase Infiltration. *Langmuir* **2020**, *36* (41), 12394–12402. https://doi.org/10.1021/acs.langmuir.0c02512.

(4) Rubinstein, M.; Colby, R. H. Ideal Chains. *Polym. Phys.* **2003**, 49–96. https://doi.org/10.1093/OSO/9780198520597.003.0002.

(5) Majoul, N.; Aouida, S.; Bessaïs, B. Progress of Porous Silicon APTES-Functionalization by FTIR Investigations. *Appl. Surf. Sci.* **2015**, *331*, 388–391. https://doi.org/10.1016/j.apsusc.2015.01.107.

(6) Lecoq, E.; Duday, D.; Bulou, S.; Frache, G.; Hilt, F.; Maurau, R.; Choquet, P. Plasma Polymerization of APTES to Elaborate Nitrogen Containing Organosilicon Thin Films: Influence of Process Parameters and Discussion About the Growing Mechanisms. *Plasma Process. Polym.* **2013**, *10* (3), 250–261. https://doi.org/10.1002/ppap.201200108.

(7) Sullivan, D. J.; O'Mahony, T. F.; Cruz-Romero, M. C.; Cummins, E.; Kerry, J. P.; Morris, M. A. The Use of Porous Silica Particles as Carriers for a Smart Delivery of Antimicrobial Essential Oils in Food Applications. *ACS Omega* **2021**, *6* (45), 30376–30385. https://doi.org/10.1021/acsomega.1c03549.
